# Phosphoglucose Isomerase Is Important for *Aspergillus fumigatus* Cell Wall Biogenesis

**DOI:** 10.1128/mbio.01426-22

**Published:** 2022-08-01

**Authors:** Yao Zhou, Kaizhou Yan, Qijian Qin, Olawale G. Raimi, Chao Du, Bin Wang, Chukwuemeka Samson Ahamefule, Bartosz Kowalski, Cheng Jin, Daan M. F. van Aalten, Wenxia Fang

**Affiliations:** a Guangxi Biological Sciences and Biotechnology Center, Guangxi Academy of Sciences, Nanning, Guangxi, China; b College of Life Science and Technology, Guangxi University, Nanning, Guangxi, China; c School of Life Sciences, University of Dundee, Dundee, United Kingdom; d State Key Laboratory of Mycology, Institute of Microbiology, Chinese Academy of Sciences, Beijing, China; University of Melbourne

**Keywords:** fungi, *Aspergillus fumigatus*, carbon flux, cell wall, drug target, phosphoglucose isomerase, structural biology

## Abstract

Aspergillus fumigatus is a devastating opportunistic fungal pathogen causing hundreds of thousands of deaths every year. Phosphoglucose isomerase (PGI) is a glycolytic enzyme that converts glucose-6-phosphate to fructose-6-phosphate, a key precursor of fungal cell wall biosynthesis. Here, we demonstrate that the growth of A. fumigatus is repressed by the deletion of *pgi*, which can be rescued by glucose and fructose supplementation in a 1:10 ratio. Even under these optimized growth conditions, the Δ*pgi* mutant exhibits severe cell wall defects, retarded development, and attenuated virulence in Caenorhabditis elegans and Galleria mellonella infection models. To facilitate exploitation of A. fumigatus PGI as an antifungal target, we determined its crystal structure, revealing potential avenues for developing inhibitors, which could potentially be used as adjunctive therapy in combination with other systemic antifungals.

## INTRODUCTION

Aspergillus fumigatus is a fungus that is widely distributed in the environment through dissemination of small, airborne conidia (1). Strong adaptability in challenging environments and dynamic response against host defenses make A. fumigatus an opportunistic pathogen, causing lung infections in immunocompromised patients (2). After being inhaled into the lung, the A. fumigatus spores germinate to hyphae, which pass through the bronchial epithelium and reach the bloodstream, leading to life-threatening infections in patients (3). A. fumigatus infections result in a morbidity rate of 50% in immunocompromised patients, with mortality rates as high as 90% even under clinical treatment (4). Recent studies suggest that 20 to 33% of patients with coronavirus disease-19 (COVID-19) were likely infected by A. fumigatus (5). The incidence of COVID-19-associated pulmonary aspergillosis (CAPA) results in a mortality over 65%, complicating the diagnosis and treatment of COVID-19 (5). To date, clinical treatments of aspergillosis have been greatly hindered by a limited arsenal of antifungal drugs (azoles, polyenes, and echinocandins) and emerging drug resistance against them (6). As such, the identification of new classes of antifungal drugs is critical for clinical treatments against invasive aspergillosis (7).

New antifungal targets are avenues for developing novel antifungal agents with new mechanisms of action. The cell wall of A. fumigatus is a dynamic network structure that comprises polysaccharides, including α-1,3-glucan, β-1,3-glucan, chitin, and galactomannan (8). Due to its essentiality to fungi and absence from animal cells, the cell wall has been considered an ideal drug target against A. fumigatus, which is exemplified by the clinical approval of echinocandins, inhibitors of β-1,3-glucan synthase that catalyzes the biosynthesis of β-1,3-glucan in the cell wall (9). Precursors for biosynthesis of cell wall polysaccharides are sugar nucleotides, including UDP-GlcNAc, UDP-Glc, and GDP-Man. Our previous studies have validated several A. fumigatus enzymes involved in sugar nucleotide biosynthesis, such as GNA1, AGM1, UAP1, and PMM, as new antifungal drug targets (10–13).

**FIG 1:**
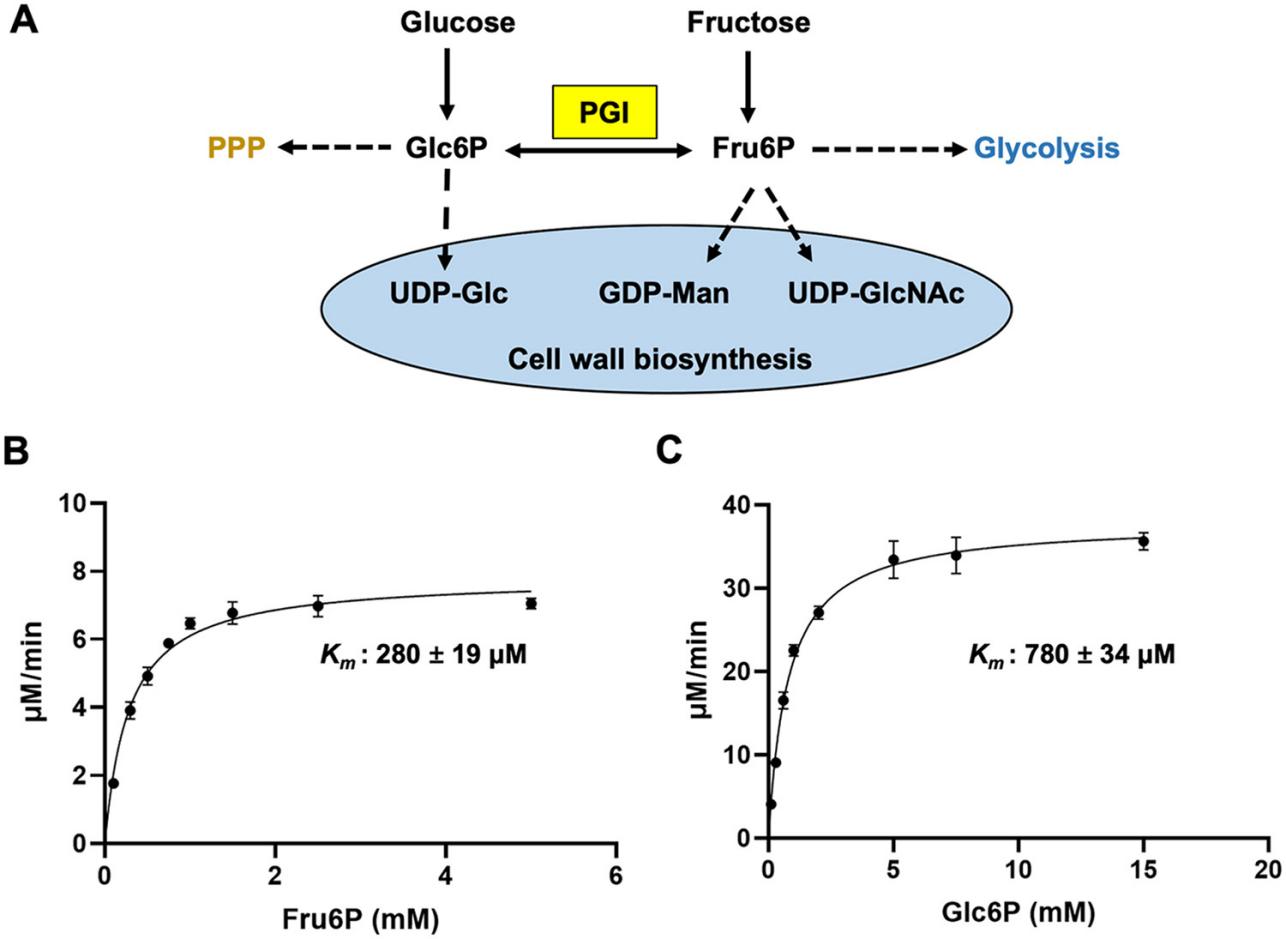
Kinetic analysis of recombinant *Af*PGI. (A) PGI-mediated cellular pathways, including PPP, glycolysis, and cell wall biosynthesis. (B) Michaelis-Menten kinetic parameters of *Af*PGI catalyzing conversion of Fru6P to Glc6P by monitoring NADPH production. First, 10 ng of PGI was added in a reaction mixture containing 50 mM HEPES (pH 7.5), 2.5 mM MgCl_2_, 0.5 mM NADP^+^, 1 mM EDTA, 0.5 U of G6PDH, and different concentrations of Fru6P in a final volume of 0.2 mL. Reaction mixtures were incubated at 30°C for 5 min and then measured by determining the emission at 460 nm and the excitation at 340 nm. The results are means ± the SD from three determinations. (C) Michaelis-Menten kinetic parameters of *Af*PGI catalyzing conversion of Glc6P to Fru6P by monitoring Fru6P production. First, 10 ng of PGI was added in a reaction mixture containing 50 mM HEPES (pH 7.5), 2.5 mM MgCl_2_, 1 mM EDTA, and different concentrations of Glc6P in a final volume of 0.2 mL, followed by incubation at 30°C for 20 min. Then, the reaction mixtures were boiled at 100°C for 10 min to stop the reactions, precipitated with ethanol at two times of volume and centrifuged. Then, 0.2 mL of the supernatant was injected, followed by monitoring by HPAEC-PAD. The results are means ± the SD from three determinations.

Phosphoglucose isomerase (PGI; EC 5.3.1.9) catalyzes the reversible conversion of fructose-6-phosphate (Fru6P) to glucose-6-phosphate (Glc6P). Therefore, PGI is involved in glycolysis and the pentose phosphate pathway (PPP). Furthermore, Glc6P can be converted to UDP-Glc, whereas Fru6P can be converted to UDP-GlcNAc and GDP-Man. These three nucleotide sugars are the main precursor molecules for all cell wall carbohydrates, including chitin (UDP-GlcNAc), galactomannan (GDP-Man), and glucan (UDP-Glc) (Fig. 1A). As such, targeting PGI is likely to affect the biosynthesis of cell wall and therefore elicits antifungal activity. Although the physiological role of PGI in A. fumigatus has not yet been explored, PGI orthologues from other Aspergillus species have been extensively studied. The PGI mutant swoM1 in A. nidulans exhibits abnormal hyphal polarity, which is reversed by replacing glucose with alternative carbon sources (14). Our recent work has demonstrated that PGI is required for the cell wall integrity of A. flavus (15). Apart from Aspergillus, the role of PGI has also been studied in other microorganisms. In Mycobacterium smegmatis, PGI disruption leads to glucose auxotrophy and defects in cell wall biosynthesis (16). Bacillus subtilis mutants lacking PGI display cell lysis in glucose and galactose, which can be prevented by adding some carbon sources such as fructose, glucosamine, or glycerol (17). PGI deficiency in Saccharomyces cerevisiae prevents full oxidation of NADPH and redirects carbon flux to the PPP pathway (18). In Cryptococcus neoformans, PGI is critical for the biosynthesis of melanin and capsule, as well as maintaining cell wall integrity and stress resistance (19). These studies demonstrate that PGI is essential for fungal carbon metabolism and cell wall biosynthesis, suggesting that PGI is a potential antifungal target against A. fumigatus.

To target PGI *in vivo* using chemical tools, small molecule inhibitors of PGI, mostly pseudo substrates such as 6-phosphogluconic acid, sorbitol-6-phosphate, ribitol-5-phosphate, erythrose-4-phosphate and 5-phosphate-d-arabinonate, have been extensively studied (20–23). The polar nature of phosphate groups reduces the membrane permeability of these inhibitors, suggesting that pseudo substrates harboring phosphate groups are not suitable compounds for modulating PGI activity *in vivo*. Limited numbers of nonphosphate inhibitors of PGIs have been reported (24). As such, chemical tools to modulate the function of PGI *in vivo* are still lacking.

Here, we have characterized A. fumigatus PGI (*Af*PGI) using multidisciplinary approaches, including genetics, structural biology, and biochemistry. This study shows that PGI is required for A. fumigatus survival and maintenance of metabolic homeostasis and possesses a potentially exploitable difference near the active site compared to the human orthologue. Taken together, this study provides a genetic and structural basis for the development of fungal PGI inhibitors.

## RESULTS

### *A. fumigatus* possesses a functional phosphoglucose isomerase.

A BLASTp search of the A. fumigatus A1163 genome database using S. cerevisiae PGI (GenBank accession No. AAA34862.1) revealed a single putative phosphoglucose isomerase (EDP54506.1) encoded by the *pgi* gene (AFUB_025630). A. fumigatus *pgi* is 1,755 bp in length and contains two exons and one intron. The *Af*PGI protein contains 553 amino acids with 71, 57, and 56% identity to Candida albicans PGI (*Ca*PGI, P83780), C. neoformans PGI (*Cn*PGI, Q5KLU5), and Homo sapiens PGI (*Hs*PGI, P06744), respectively.

To investigate whether the putative *Af*PGI possesses phosphoglucose isomerase activity, we overexpressed full-length *Af*PGI with an N-terminal glutathione *S*-transferase (GST) fusion tag in Escherichia coli. After purification using glutathione beads, GST tag cleavage by PreScission Protease and size exclusion chromatography, pure *Af*PGI (9 mg/L) was obtained. A coupled assay with Glc6P dehydrogenase (G6PDH) was used to determine kinetic parameters of the catalysis of Fru6P to Glc6P. High-performance anion-exchange chromatography with pulsed amperometric detection (HPAEC-PAD) was used to evaluate the reverse reaction. The *K_m_* value of *Af*PGI for Fru6P is 280 ± 19 μM (Fig. 1B), which is similar to that of *Hs*PGI (25). The catalytic efficiency *k*_cat_/*K_m_* is 0.57 μM^−1^ s^−1^, which is approximately one-third of that of *Hs*PGI (25). The *K_m_* value of *Af*PGI for Glc6P is 780 ± 34 μM with *k*_cat_/*K_m_* = 0.99 μM^−1^ s^−1^ (Fig. 1C), suggesting that *Af*PGI-mediated Glc6P to Fru6P conversion is at a comparable efficiency as the forward reaction. Taken together, these data show that A. fumigatus possesses a functional phosphoglucose isomerase.

### *pgi* is important for survival of *A. fumigatus*.

To investigate the physiological role of *pgi* in A. fumigatus, a knockout construct was created with a *neo-AnpyrG-neo* selection cassette flanked by upstream and downstream fragments (see Fig. S1A in the supplemental material). Because a Δ*pgi* mutant may not be viable, we attempted to complement with products of PGI during screening (Fru6P/Glc6P; 1% fructose and 1% glucose were supplemented as their sugar precursors in the screening media). Δ*pgi* clones were obtained and confirmed by PCR with multiple pairs of primers and Southern blotting (see Fig. S1B and C). To construct the revertant strain (RT), the confirmed Δ*pgi* mutant was plated on 5-fluoroorotic acid (5-FOA) to obtain the Δ*pgi *Δ*pgrG* strain. The revertant construct with *AfpyrG* inserted after the *pgi* gene was transformed into the Δ*pgi *Δ*pgrG* protoplasts. PCR and Southern blotting were applied for the wild-type (WT), Δ*pgi*, Δ*pgi *Δ*pgrG*, and RT strains (see Fig. S1 and Table S1).

**FIG 2:**
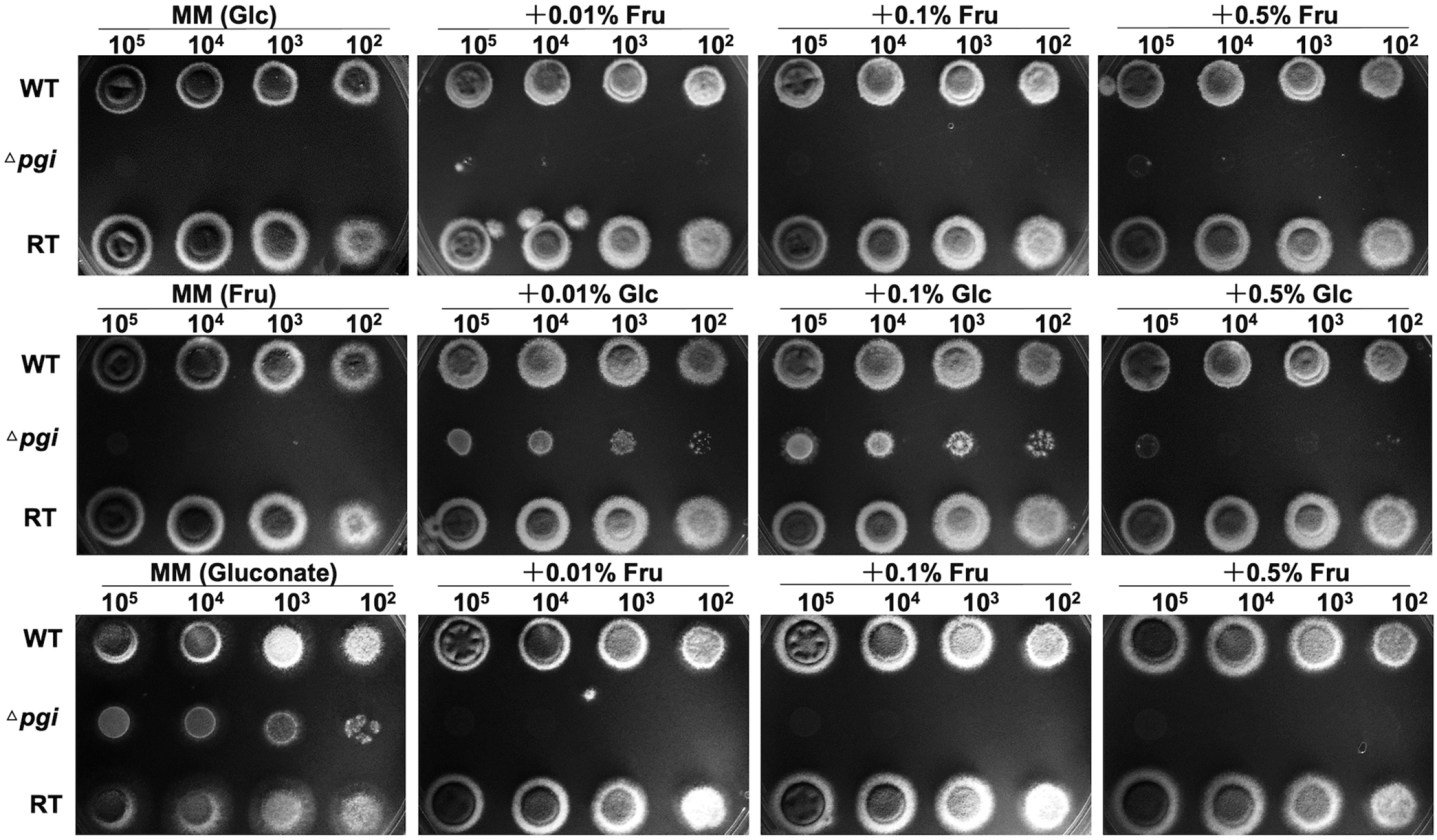
Growth of the Δ*pgi* mutant in different carbon sources. Conidia at 10^2^ to 10^5^ from the WT, Δ*pgi*, and RT strains were grown on MM with combinations of (i) 1% Glc with 0.01 to 0.5% Fru, (ii) 1% Fru with 0.01 to 0.5% Glc, or (iii) 1% gluconate with 0.01 to 0.5% Fru for carbon sources. Plate photos were taken after cultivation at 37°C for 48 h.

*pgi* mutants in different species have requirements for different carbon sources (14, 16, 26). Therefore, we tested the growth of the Δ*pgi* mutant on plates supplemented with a range of carbon sources. The Δ*pgi* mutant could not grow when 1% Glc was the sole carbon source, in agreement with the fact that it also could not grow when the sole carbon source was 1% maltose (see Fig. S2), which can be hydrolyzed to glucose by amylase. The addition of 1 to 5% Fru gradually restored growth, with 10% Fru supporting maximal growth (Fig. 2; see also Fig. S2), but the strain was not viable with 1% Fru as the sole carbon source. Addition of 0.01% Glc partially restored growth and 0.1% Glc recovered growth to the best level. However, 0.5% Glc fully suppressed growth and 1% to 10% Glc partially restored mutant growth (Fig. 2; see also Fig. S2A). These results suggest a 10:1 ratio of Fru to Glc is optimal to support growth of the Δ*pgi* mutant, which is different from the requirement (Fru/Glc ratio of 2:1) of the Δ*pgi* mutant in A. flavus (15). Interestingly, 1% gluconate supported the growth of the Δ*pgi* mutant but the addition of 0.01 to 0.5% Fru disrupted the growth (Fig. 2). The other sole carbon sources, 1% mannose and 1% glucosamine, did not support growth of the mutant, whereas 1% galactose, 1% GlcNAc, and 1% glycerol partially supported growth (see Fig. S2B). These observations support the role of *Af*PGI as a key node at the intersection of metabolic pathways. These results also demonstrate that viability of the Δ*pgi* mutant can be restored by the substrate (Glc6P) and product (Fru6P), suggesting that the enzyme activity of PGI is important for A. fumigatus. Taken together, these results demonstrate that *pgi* is important for A. fumigatus survival.

10.1128/mbio.01426-22.1 FIG S1 Generation of Δ*pgi* mutant. (A) Diagram illustrating strategies for constructing Δ*pgi* mutant and RT strains. (B) Southern blot analysis using total genomic DNA digested with BglII and NcoI and probed with downstream fragment of *pgi* and *AnpyrG*. The Δ*pgi* Δ*pgrG* strain was simply labeled as Δ′. (C) Confirmation of the mutant and revertant strains by PCR using the primer pairs P1/P2, P3/P4, P5/P6, P7/P8, and P9/P10, as shown in the figure. The Δ*pgi* Δ*pgrG* strain was labeled as Δ′. Download FIG S1, PDF file, 0.1 MB. Copyright © 2022 Zhou et al. 2022 Zhou et al. https://creativecommons.org/licenses/by/4.0/ This content is distributed under the terms of the Creative Commons Attribution 4.0 International license.

10.1128/mbio.01426-22.2 FIG S2 Growth of the Δ*pgi* mutant in different carbon sources. (A) 10^2^ to 10^5^ conidia of the WT, Δ*pgi*, and RT strains were inoculated onto MM medium with combinations of (i) 1% Glc with 1 to 10% Fru or (ii) 1% Fru with 1 to 10% Glc. Plate photos were taken after cultivation at 37°C for 48 h. (B) Sole carbon sources such as 1% galactose (Gal), 1% maltose (Mal), 1% mannose (Man), 1% glucosamine (GlcN), 1% *N*-acetylglucosamine (GlcNAc), or 1% glycerol were used for growth tests. Plate photos were taken after cultivation at 37°C for 48 h. Download FIG S2, PDF file, 0.2 MB. Copyright © 2022 Zhou et al. 2022 Zhou et al. https://creativecommons.org/licenses/by/4.0/ This content is distributed under the terms of the Creative Commons Attribution 4.0 International license.

10.1128/mbio.01426-22.7 TABLE S1 Primers used in this study. Download Table S1, PDF file, 0.1 MB. Copyright © 2022 Zhou et al. 2022 Zhou et al. https://creativecommons.org/licenses/by/4.0/ This content is distributed under the terms of the Creative Commons Attribution 4.0 International license.

### *pgi* deletion leads to reduced cell growth, decreased conidiation, and delayed germination.

**FIG 3:**
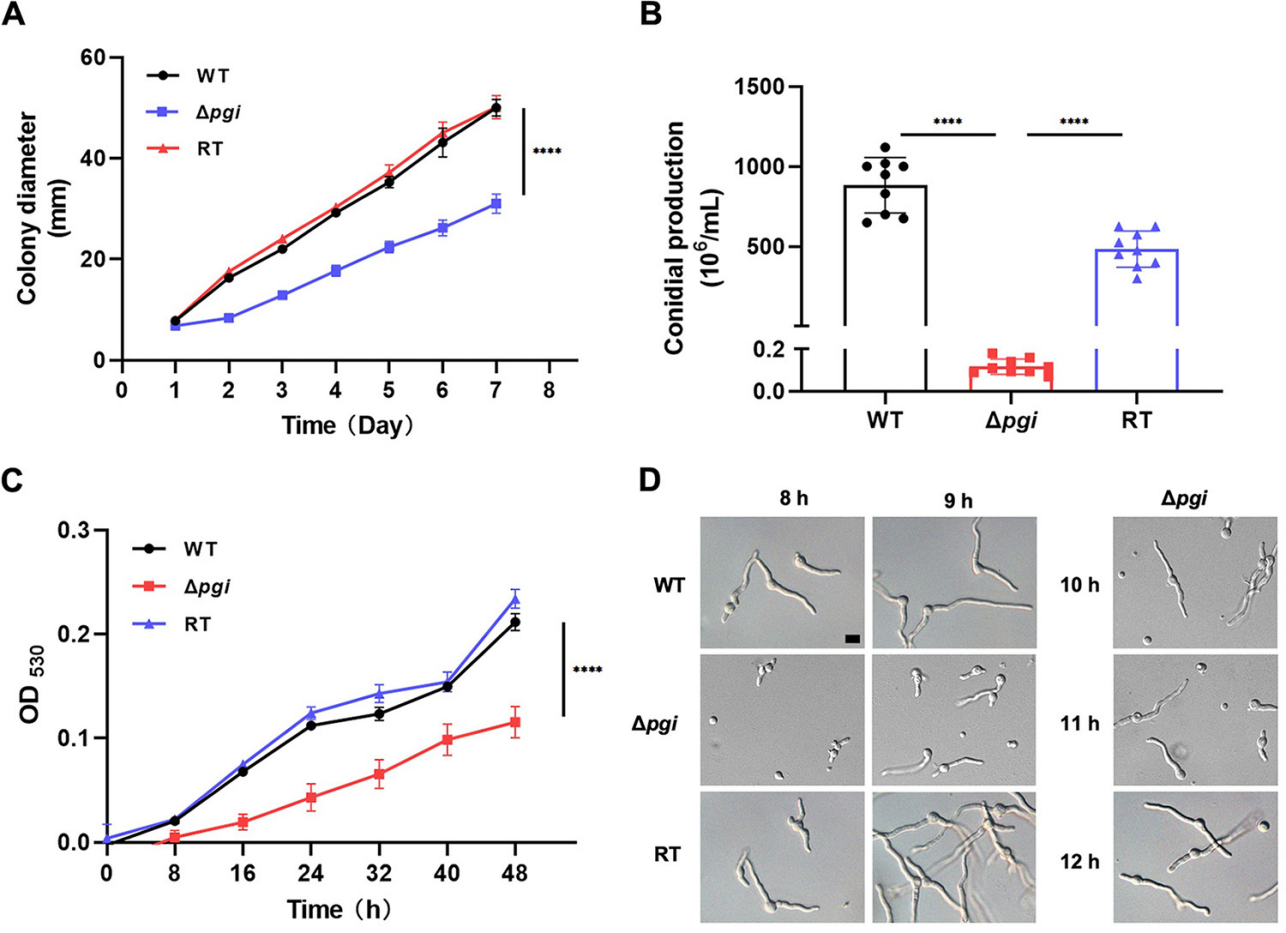
Growth phenotypes of the Δ*pgi* mutant under MMFG cultivation. (A) A total of 2 × 10^5^ conidia from the WT, Δ*pgi*, and RT strains were inoculated onto solid MMFG media, and colony diameters were measured daily for 7 days at 37°C cultivation. Data represent means ± the SD from three replicates. (B) After 7 days of cultivation, conidia were harvested and counted by using a hemocytometer. Values represent means ± the SD, a multiple *t* test analysis was applied, and the *P* value indicates statistical significance (****, *P* < 0.0001). (C) The growth rate in liquid MMFG was continuously recorded by plate reader for 48 h at 30°C. Values represent means ± the SD from three replicates. (D) Germination was examined by using a differential interference contrast microscope (Leica) for the indicated time points at 37°C cultivation. Scale bar, 10 μm.

To study the role of *Af*PGI, a minimal medium containing 1% Fru and 0.1% Glc (MMFG) was used for the subsequent experiments to support growth of the mutant. Solid MMFG plates were used to evaluate daily expansion of colony diameter. The Δ*pgi* mutant displayed significantly reduced colony growth compared to the WT or RT strain (Fig. 3A). The numbers of conidia produced by the mutant were >7,000- and >4,000-fold less than the WT and RT strains, respectively (*P < *0.0001, unpaired *t* test; Fig. 3B). The growth rate in liquid MMFG also revealed that the Δ*pgi* mutant grew slower than the WT or RT strain (Fig. 3C), which is consistent with the growth on solid MMFG. Inspection of the germination rate at the early stage under the microscope revealed that the WT or RT strain reached 97% germination after 8 h of incubation, whereas the Δ*pgi* mutant had only 40% germination at 8 h and 72% germination at 12 h (Fig. 3D; see also Table S2), but growth polarity was not affected, which is different from the *pgi* mutant (swoM1) in A. nidulans (14). We note a fraction of ungerminated spores in the mutant compared to the WT strain, but we have not investigated the mechanism behind this. Taken together, these results demonstrate that *pgi* deletion in A. fumigatus leads to reduced cell growth, decreased conidiation and delayed germination.

10.1128/mbio.01426-22.8 TABLE S2 Germination rates of the three strains in liquid MMFG. Download Table S2, PDF file, 0.2 MB. Copyright © 2022 Zhou et al. 2022 Zhou et al. https://creativecommons.org/licenses/by/4.0/ This content is distributed under the terms of the Creative Commons Attribution 4.0 International license.

### *pgi* deletion affects cell wall integrity.

**FIG 4:**
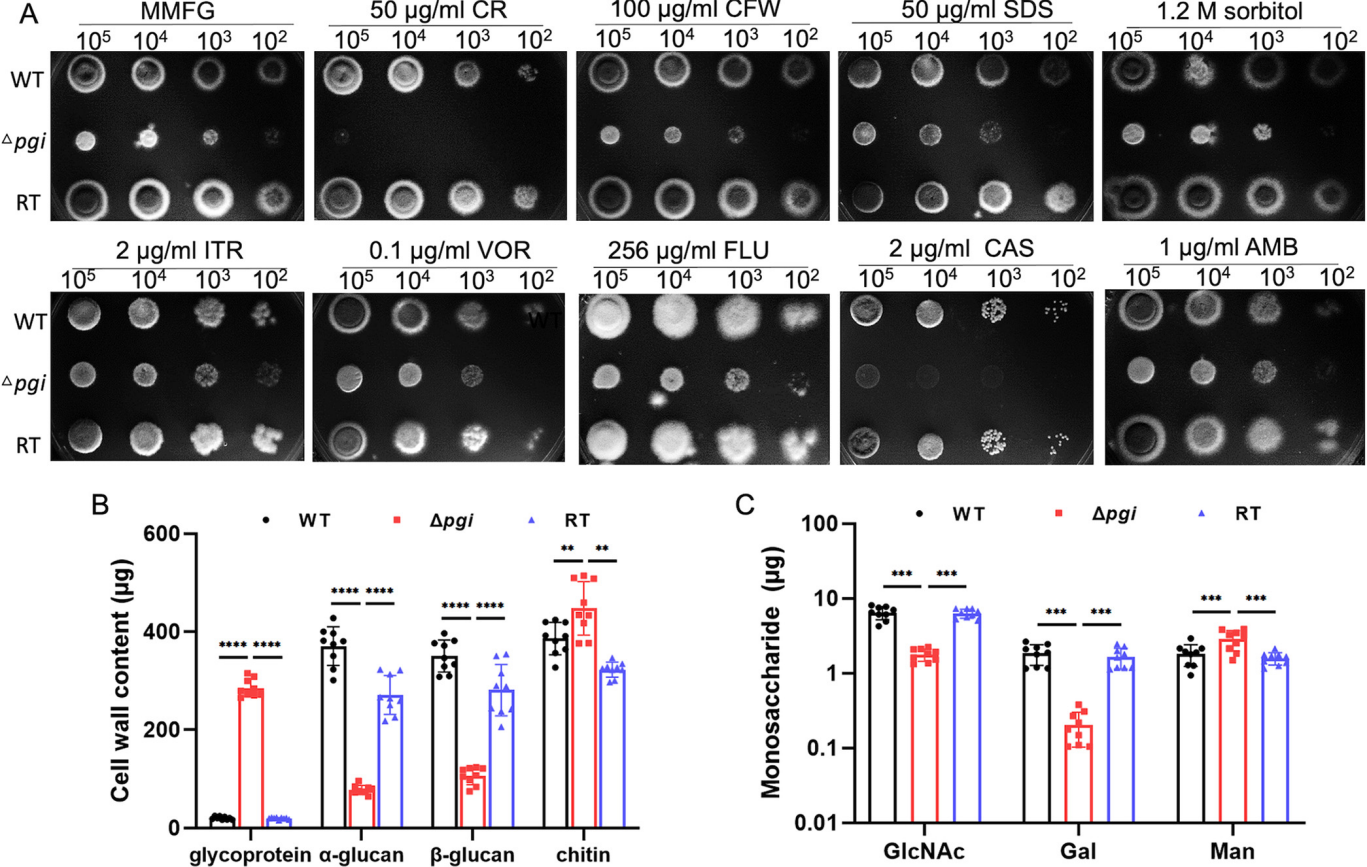
Cell wall integrity and component analysis of the Δ*pgi* mutant. (A) Sensitivity of the Δ*pgi* mutant to chemical agents and antifungal drugs. Serial dilutions of conidia were inoculated onto solid MMFG media supplemented with the indicated chemical agents or drugs and then incubated at 37°C for 48 h. (B) A total of 1 × 10^8^ conidia were inoculated into 100 mL of liquid MMFG at 37°C for 48 h, and 10 mg of lyophilized mycelia was used to quantify the cell wall contents. Values represent means ± the SD from three replicates, and multiple *t* tests were applied to calculate the *P* values. (C) Monosaccharides from cell wall glycoproteins were released by acid hydrolysis and then quantified by HPAEC-PAD. Values represent means ± the SD from three replicates, and multiple *t* tests were applied to calculate the *P* values.

Since PGI is likely to affect precursors of cell wall carbohydrates (UDP-Glc, UDP-GlcNAc, and GDP-Man), deletion of *pgi* may affect the cell wall integrity. To validate this hypothesis, we tested here the sensitivity of the Δ*pgi* mutant toward cell wall disrupting agents such as Calcofluor White, Congo red (CR), the cell membrane perturbing agent SDS, the osmotic agent sorbitol, and antifungal drugs affecting the plasma membrane (itraconazole [ITR], voriconazole [VOR], fluconazole [FLU] and amphotericin B [AMB]) and cell wall (caspofungin [CAS]). As shown in Fig. 4A, the Δ*pgi* mutant was sensitive to low concentrations of CR and CAS, suggesting that β-glucan synthesis may be affected in the Δ*pgi* mutant. Analysis of the cell wall components revealed that the α/β-glucan content of the mutant was reduced by 70 to 79%, whereas the content of chitin was increased by 16% together with a 14-fold increase in glycoprotein content (Fig. 4B). Although the cell wall glycoprotein content was significantly increased, the GlcNAc and Gal contents released from these proteins were reduced by 72 and 89%, respectively, while the released Man was increased by 56% in the mutant (Fig. 4C; see also Table S3A). Collectively, these results suggest that deletion of *pgi* remarkably affects the synthesis of cell wall glucan in A. fumigatus and triggers a compensatory increase of chitin and glycoprotein. Therefore, *pgi* deletion affects cell wall integrity.

10.1128/mbio.01426-22.9 TABLE S3 Monosaccharides and intracellular sugars in the three strains. Download Table S3, PDF file, 0.1 MB. Copyright © 2022 Zhou et al. 2022 Zhou et al. https://creativecommons.org/licenses/by/4.0/ This content is distributed under the terms of the Creative Commons Attribution 4.0 International license.

### *pgi* deletion affects homeostasis of intracellular nucleotide sugars and phosphate sugars.

**FIG 5:**
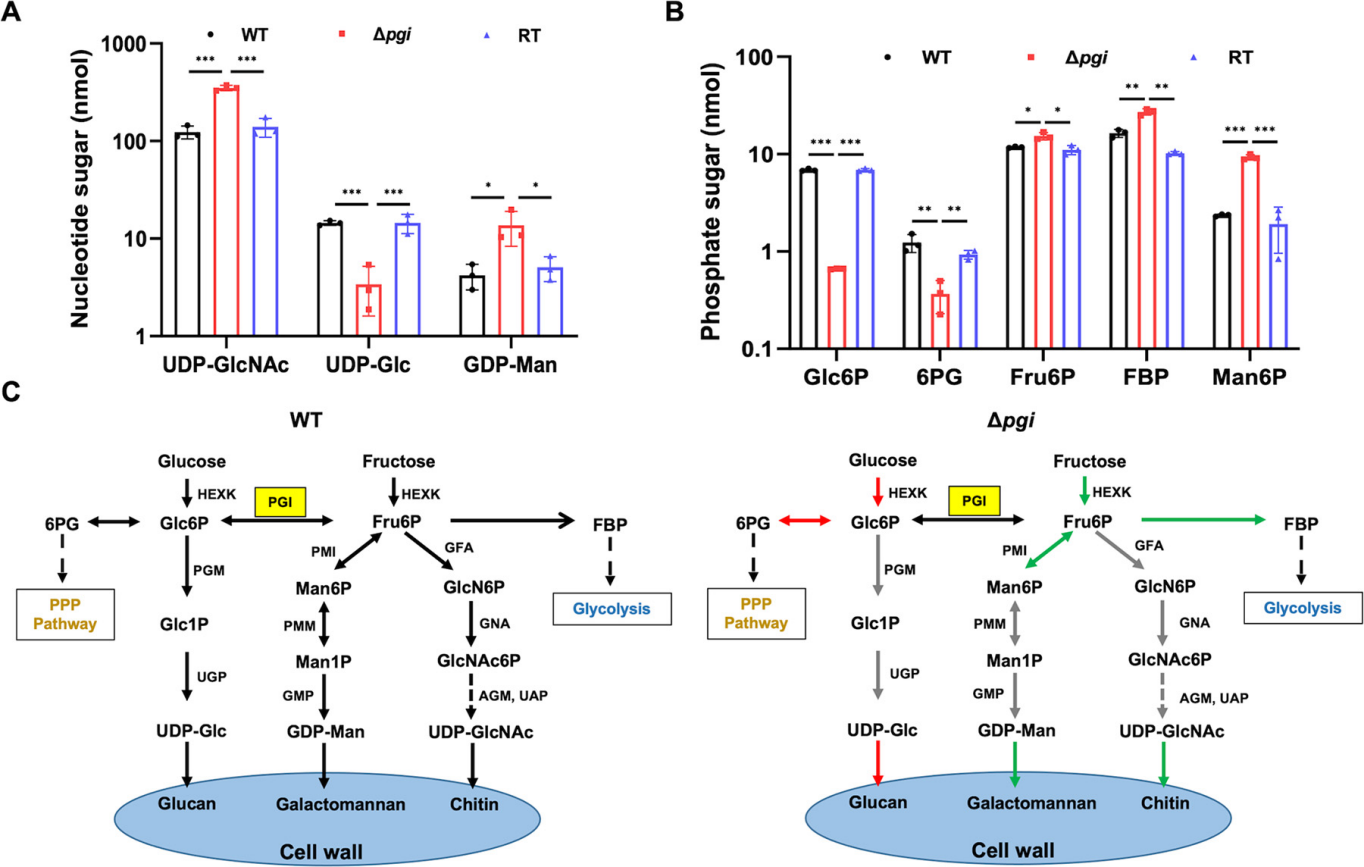
Effect of *pgi* deletion on the homeostasis of intracellular nucleotide sugars and phosphate sugars. A total of 1 × 10^8^ conidia were inoculated into 100 mL of liquid MMFG medium for cultivation at 37°C for 48 h, and mycelia were flash-frozen and lyophilized. Intracellular metabolites were extracted from 50 mg of dry mycelia from each strain. Three biological replicates were performed, and multiple *t* tests were used to calculate the *P* values. (A) Nucleotide sugars from extracted metabolites were detected by HPLC with a CarboPac PA-1 column at UV 256 nm detection. (B) Phosphate sugars from extracted metabolites were detected by HPEAC-PAD with a CarboPac PA-1 column. (C) Metabolic flux comparison between WT and Δ*pgi* mutant strains. Green or red arrows indicate increases or decreases, respectively.

Because the cell wall contents were altered in the Δ*pgi* strain, we next used a high-pressure liquid chromatography (HPLC) approach to trace the levels of intracellular nucleotide sugars (UDP-Glc, UDP-GlcNAc, and GDP-Man), the precursors of cell wall polysaccharides, after growth in MMFG media (Fig. 5A; see also Table S3B). Compared to the WT, the amount of UDP-Glc in the mutant was reduced by 77% (*P < *0.001, 14.5 ± 0.8 nmol in the WT versus 3.4 ± 1.8 nmol in the mutant), while the amount of UDP-GlcNAc was increased by 185% (*P < *0.001, 124 ± 19 nmol in the WT versus 355 ± 22 nmol in the mutant), and the GDP-Man amount was increased by 225% (*P < *0.05, 4.2 ± 1.2 nmol in the WT versus 13.7 ± 5.3 nmol in the mutant) (Fig. 5A; see also Table S3B). These results suggest that 1% Fru in MMFG media provides sufficient amounts of sugar to synthesize UDP-GlcNAc and GDP-Man for the Δ*pgi* strain, while 0.1% Glc in MMFG medium is unable to restore the intracellular content of UDP-Glc back to a normal level, causing a defect in glucan synthesis. Taken together, these data show that *Af*PGI plays a key role in maintaining the level of intracellular nucleotide sugars (UDP-Glc, UDP-GlcNAc, and GDP-Man) in A. fumigatus (Fig. 5C).

Phosphate sugars are the building blocks for nucleotide sugar biosynthesis (27). To dissect the mechanisms underpinning the changes in sugar nucleotide concentrations, the amounts of intracellular phosphate sugars were determined by HPAEC-PAD. Compared to the WT, the amounts of Glc6P and 6-phosphogluconic acid (6PG) in the Δ*pgi* mutant were reduced by 90% (*P < *0.001, 6.9 ± 0.2 nmol in the WT versus 0.66 ± 0.01 nmol in the mutant) and 70% (*P < *0.01, 1.2 ± 0.3 nmol in the WT versus 0.4 ± 0.1 nmol in the mutant), respectively (Fig. 5B; see also Table S3C). In the mutant, Fru6P was increased by 30% (*P < *0.05, 11.8 ± 0.2 nmol in the WT versus 15.3 ± 1.3 nmol in the mutant), Man6P was increased by 295% (*P < *0.001, 2.4 ± 0.1 nmol in the WT versus 9.3 ± 0.6 nmol in the mutant), and fructose-1,6-biphosphate (FBP) was increased by 65% (*P < *0.01, 16.4 ± 1.6 nmol in the WT versus 27.2 ± 2.0 nmol in the mutant) (Fig. 5B; see also Table S3C). These data show that *Af*PGI is a key enzyme in maintaining the homeostasis of intracellular phosphate sugars (Fig. 5C).

### *pgi* deletion leads to attenuated virulence in nematode and larva infection models.

**FIG 6:**
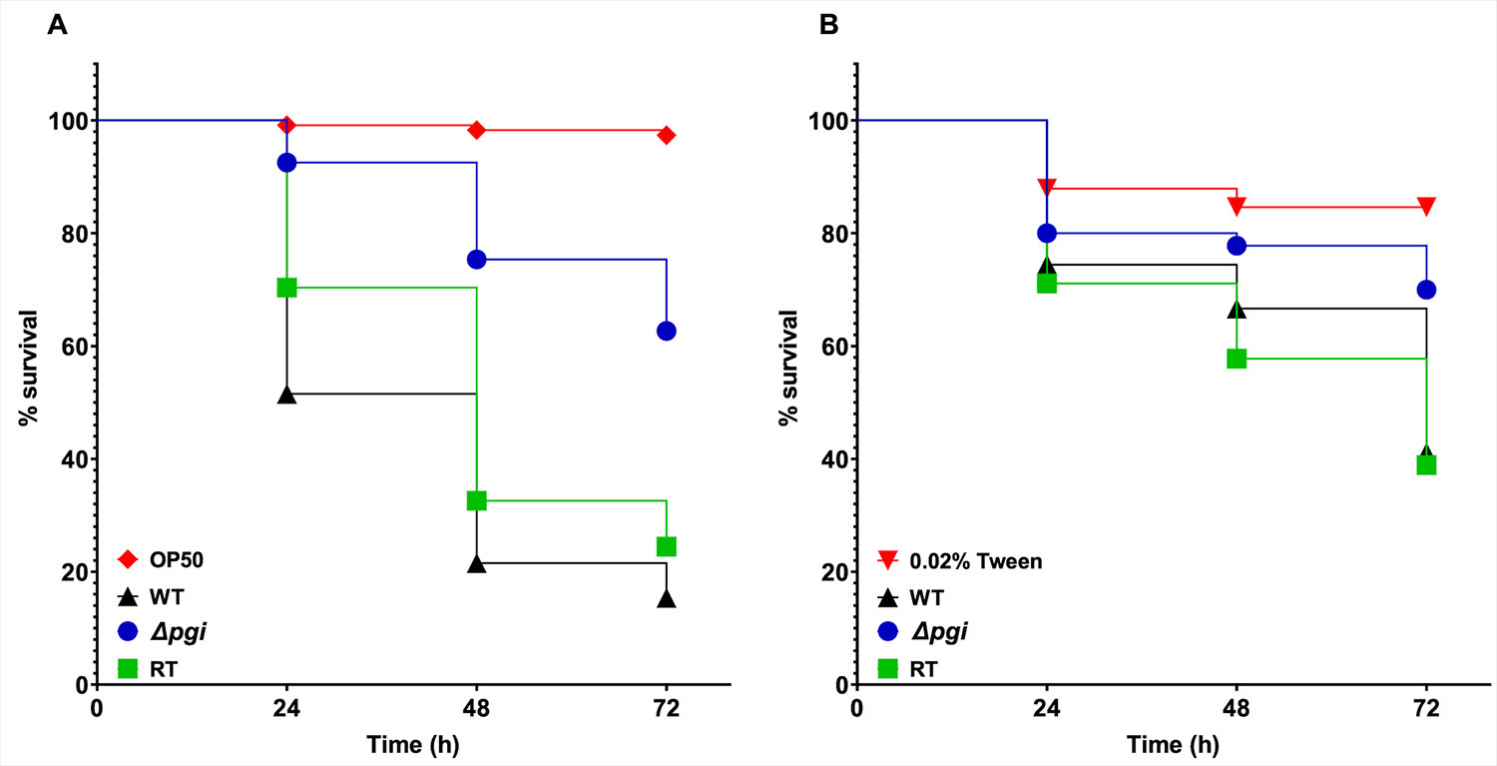
Effect of *pgi* deletion on A. fumigatus virulence. (A) Kaplan-Meier curves were plotted to show the survival rates of *glp-4(bn2)*; *sek-1(km4)* C. elegans nematodes after 16 h preinfection with conidia of the indicated A. fumigatus strains. Three biological repeats (each with triplicates) were conducted for each strain. OP50 is the E. coli food for the nematodes. (B) Kaplan-Meier curves of the survival rate of G. mellonella larvae at 24, 48, and 72 h after conidial injection. Three biological repeats with triplicates were applied for each strain.

To evaluate the effect of *pgi* deletion on virulence of A. fumigatus, a recently developed Caenorhabditis elegans-based A. fumigatus infection model was applied (28). Survival rates of *glp-4(bn2)*; *sek-1(km4)* worms infected by the WT, Δ*pgi* mutant, and RT strains at 24, 48, and 72 h were recorded and plotted as Kaplan-Meier survival curves. As shown in Fig. 6, the Δ*pgi* mutant exhibited a significantly attenuated virulence (*P < *0.0001) with a survival rate of 63% ± 4% at 72 h (Fig. 6A; see also Table S4A). The hyphal filamentation rate of the mutant at 24 h was 16% ± 8%, lower (*P < *0.0001) than the rates in the WT and RT strains (64% ± 4% and 45% ± 3% for the WT and RT strains, respectively; see Table S4A).

10.1128/mbio.01426-22.10 TABLE S4 Survival rates of the nematodes and G. mellonella larvae infected by the indicated strains. Download Table S4, PDF file, 0.1 MB. Copyright © 2022 Zhou et al. 2022 Zhou et al. https://creativecommons.org/licenses/by/4.0/ This content is distributed under the terms of the Creative Commons Attribution 4.0 International license.

To further evaluate the virulence of the Δ*pgi* mutant, a previously used Galleria mellonella model was applied (10). A Kaplan-Meier survival curve was plotted for dead larvae infected by A. fumigatus strains. Like the result in the nematode model, the survival rate of the Δ*pgi* mutant was higher than those of the WT and RT strains after 72 h of injection, revealing significant attenuated virulence of the mutant (*P* < 0.01, Fig. 6B; see also Table S4B). Taken together, these data show that *pgi* deletion leads to attenuated virulence in nematode and larva infection models.

### The crystal structure of *Af*PGI reveals avenues for discovery of inhibitors.

The data with the mutant strain suggests that *Af*PGI is important for cell wall biosynthesis and the survival of A. fumigatus. To support exploitation of *Af*PGI as an antifungal target, we next determined the crystal structure of *Af*PGI and compared it to its human orthologue. Although structures of PGI have been extensively studied (29–31), no structure of a fungal PGI has been reported to date. Purified recombinant *Af*PGI from E. coli was crystallized in HEPES and tri-sodium citrate solutions in the presence or absence of Glc6P, and high-resolution synchrotron data were collected (Table 1). Using the structure of porcine PGI (PDB code 1GZD [32]) as a search model, the *Af*PGI structure was solved by molecular replacement with one molecule per an asymmetric unit and refined to 1.78 Å with an *R*_work_ value of 15.4 and an *R*_free_ value of 18.0 (Table 1). *Af*PGI was also crystallized in the same condition without Glc6P, and the apo structure of *Af*PGI was solved by molecular replacement using the *Af*PGI-Glc6P complex structure as the search model and refined to 1.56 Å with an *R*_work_/*R*_free_ value of 15.7/18.3 (Table 1).

**TABLE 1:** Summary of data collection and structure refinement statistics^*a*^

Parameter	*Af*PGI-Glc6P complex	*Af*PGI
Space group	P4_3_2_1_2	P4_3_2_1_2
Unit cell dimensions		
*a*, *b*, *c* (Å)	85.3, 85.3, 232.5	84.5, 84.5, 232.0
*R*_merge_ ^*b*^	0.095 (1.29)	0.13 (1.716)
CC_1/2_ (%)	99.9	100
Completeness (%)	99.6 (99.3)	95.8 (82.5)
Redundancy	6.5 (6.6)	26.4 (22.5)
Resolution range (Å)	48.03–1.78 (1.84–1.78)	79.36–1.56 (1.75–1.56)
No. of observations	536,192 (52,715)	1,885,405 (80,273)
No. of unique observations	82,761 (7,939)	71,374 (3,567)
*R* (%)^*c*^	15.4	15.7
*R*_free_ (%)^*c*^	18.0	18.3
*I*/σ〈*I*〉	11.4 (1.5)	19.7 (2.0)
No. of atoms		
Protein	4,437	8,565
Glc6P	16	
Water	495	459
Glycerol	30	42
Sodium ion	1	4
Chlorin ion	2	2
Citrate		18
B-factors (Å^2^)		
Protein	29.4	21.7
Glc6P	42.9	
Water	39.2	28.4
Glycerol	57.4	40.2
Sodium ion	29.8	25.2
Chlorin ion	63.5	53.6
Citrate		56.3
RMSD from ideal geometry		
Bond length (Å)	0.012	0.010
Bond angle (°)	1.7	1.1
PDB code	7OYL	7U34

a Values of the outer shell are shown in parentheses.

b $R_{\text{merge}}=\frac{\sum_{\mathrm{hkl}}\sum_{i=1}^{n}|I_{i}\left(\mathrm{hkl}\right)-\bar{I}\left(\mathrm{hkl}\right)|}{\sum_{\mathrm{hkl}}\sum_{i=1}^{n}I_{i}\left(\mathrm{hkl}\right)}$. *I_i_* represents the observed intensity, and *I* represents the average intensity.

c $R=\frac{\sum_{\mathrm{hkl}}\left|F_{\text{obs}}\left(\mathrm{hkl}\right)-F_{\text{cal}}\left(\mathrm{hkl}\right)\right|}{\sum_{\mathrm{hkl}}F_{\text{obs}}\left(\mathrm{hkl}\right)}$. *F*_obs_ represents the observed structure factor amplitude. *F*_cal_ represents the calculated structure factor amplitude. *R*_free_ was calculated using 5% of *F*_obs_ values that have not been used for building model.

**FIG 7:**
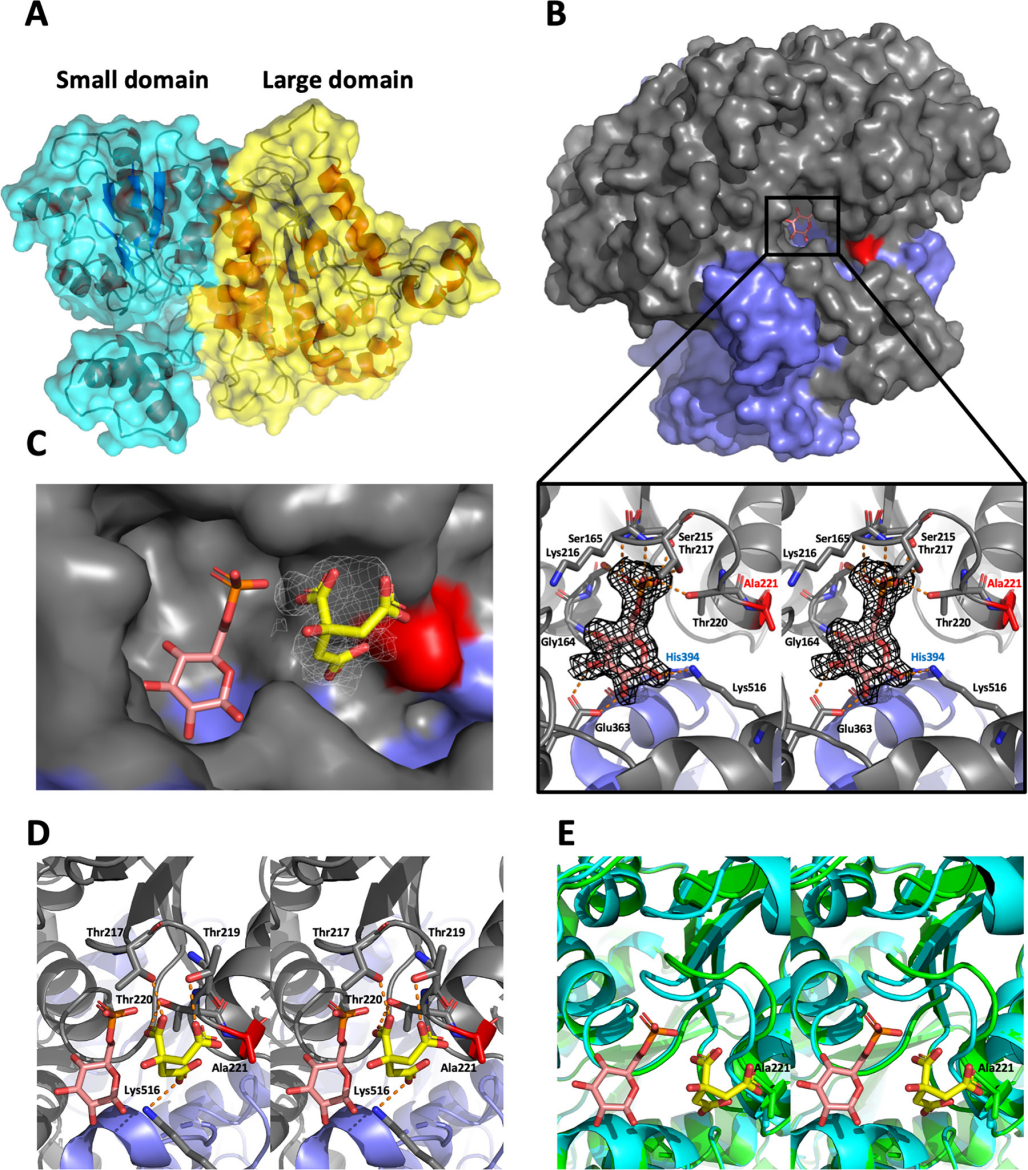
Crystal structure of *Af*PGI in complex with Glc6P. (A) Overall structure of the *Af*PGI monomer. Large domain and small domains are depicted in yellow and cyan, respectively. (B) Structure of the dimeric form of *Af*PGI, generated by crystallographic symmetry. The overall structure of *Af*PGI is shown as a surface representation. Two monomers are color gray and blue, respectively. Orange sticks indicate the Glc6P molecule in the active site. The red surface represents Ala221 that is not conserved in *Hs*PGI. The closeup view shows the active site of *Af*PGI. Orange dots indicate hydrogen bonds. The black mesh around the Glc6P molecule indicates the *F_o_*-*F_c_* map (contoured at 2.5σ) before inclusion of the ligand. (C) Closeup view of the active site of apo *Af*PGI (surface representation). Two monomers are shown in gray and blue, respectively. Red represents the nonconserved residue A221. Glc6P (orange sticks) was placed by superimposing of the structure of *Af*PGI-Glc6P complex onto that of apo *Af*PGI. White meshes represent the unbiased *F_o_*-*F_c_* map (contoured at 2.5σ) around a citrate molecule (yellow sticks). (D) Ribbon representation of panel C. Residues recognizing citrate are shown as sticks. Hydrogen bonds are shown as orange dots. (E) Superposition of the structure of apo *Af*PGI (green) onto that of *Af*PGI-Glc6P complex (cyan). Yellow sticks represent citrate.

The structure of the *Af*PGI monomer in the asymmetric unit contains a large domain and a small domain (Fig. 7A), similar to that of *Hs*PGI (PDB code 1IRI [33]), with a root mean square deviation (RMSD) of 0.4 Å on Cα atoms. Each domain exhibits an α/β fold in which a central β-sheet is flanked by α-helixes (Fig. 7A). A physiological dimer is formed by crystallographic symmetry (Fig. 7B, 5,904-Å^2^ buried surface area). During refinement, an unbiased *F_o_*-*F_c_* density was observed in the interface between two protein monomers and resolved as a Glc6P molecule (Fig. 7B). The phosphate recognition site is a loop consisting of Ser215, Lys216, Thr217, and Thr220 (Fig. 7B). Ser165 also forms a hydrogen bond with the phosphate group. Hydroxyl groups on the sugar ring form hydrogen bonds to Gly164, Glu363, His394, and Lys516 (Fig. 7B). Sequence alignment shows that these residues are conserved in all PGI orthologues and contribute to a conserved reaction mechanism that has been extensively discussed (29–31, 34, 35). To date, inhibitors against PGI are limited to pseudo substrates harboring phosphate groups (32, 34, 36, 37). These compounds have negatively charged phosphate groups linked to low membrane permeability and therefore are not suitable as chemical tools for probing *Af*PGI function *in vivo*. Most proteins recognize phosphate groups by salt bridges with His/Lys/Arg side chains (38–40). Strikingly, *Af*PGI only exploits the protein backbone or neutral side chains to interact with the substrate phosphate group (Fig. 7B). Previous studies have shown that phosphate groups can be mimicked by neutral scaffolds (41). For instance, a neutral diphosphate mimic has been demonstrated to inhibit the activity of human O-GlcNAc transferase (OGT), an enzyme that recognizes UDP-GlcNAc harboring a diphosphate group (42). By docking the neutral diphosphate mimic into the active site of *Af*PGI, we observed that this compound is predicted to interact with the phosphate-binding site via two carbonyl groups (see Fig. S3), which is similar to the predicted binding mode in OGT (42), suggesting that neutral phosphate mimics could bind to the active site of *Af*PGI. Therefore, neutral phosphate mimics, substituting for phosphate groups of pseudo substrates, may reduce their polarity, enhance the membrane permeability, and in turn act as *Af*PGI inhibitors.

10.1128/mbio.01426-22.3 FIG S3 Prediction of the binding mode of a neutral bisphosphate mimic in the active site of *Af*PGI. (A) Structure of the neutral bisphosphate mimic. (B) Predicted binding mode of the neutral bisphosphate mimic (green sticks) in the *Af*PGI active site (cyan). Prediction was carried out by AutoDock using the *Af*PGI-Glc-6P structure as the macromolecule for docking. Representative binding mode of the neutral bisphosphate mimic. Orange dots indicate hydrogen bonds. Download FIG S3, PDF file, 0.3 MB. Copyright © 2022 Zhou et al. 2022 Zhou et al. https://creativecommons.org/licenses/by/4.0/ This content is distributed under the terms of the Creative Commons Attribution 4.0 International license.

*Hs*PGI is essential for vertebrate embryo development (43, 44), and therefore selectivity is an important factor in the development of *Af*PGI inhibitors. While the residues directly involved in catalysis are conserved, *Af*PGI Ala221, at the end of the phosphate-binding loop and 9 Å away from Glc6P (Fig. 7B), is substituted with a bulky Gln (Q216) in *Hs*PGI. Sequence alignment shows that Ala221 is conserved in several pathogenic fungi, including Candida albicans, Stachybotrys chartarum, and Histoplasma capsulatum (see Fig. S4). In the structure of the *Af*PGI-Glc6P complex, there is a gap (6 Å) between Ala221 and Gln523 (corresponding to Glu526 in *Hs*PGI) (see Fig. S5). As such, the side chain amine of Lys520 (corresponding to Lys523 in *Hs*PGI) is placed into this gap. The intrinsic flexibility of lysine implies that the Lys520 sidechain is likely to move away from the gap, in agreement with the fact that there is no electron density around the Lys520 sidechain in the structure of apo *Af*PGI. This intrinsic mobility of Lys520 could leave an empty gap between Ala221 and Gln523. In contrast, for the counterparts of *Af*PGI Ala221/Gln523 in *Hs*PGI (Gln216/Gln526), a hydrogen bond between Gln216 and Glu526, and a salt bridge between Glu526 and Lys523 occupy this gap.

10.1128/mbio.01426-22.4 FIG S4 Partial sequence alignment of PGIs. Red triangle indicates Ala221 in *Af*PGI. Sequences are from GenBank with the codes EDP54506.1 (A. fumigatus), XP_713513.1 (Candida albicans), XP_018229466.1 (Pneumocystis jirovecii), KEY64212.1 (Stachybotrys chartarum), QSS62647.1 (Histoplasma capsulatum), XP_569228.1 (Cryptococcus neoformans), and NP_000166.2 (Homo sapiens). Download FIG S4, PDF file, 0.1 MB. Copyright © 2022 Zhou et al. 2022 Zhou et al. https://creativecommons.org/licenses/by/4.0/ This content is distributed under the terms of the Creative Commons Attribution 4.0 International license.

10.1128/mbio.01426-22.5 FIG S5 Structures of *Af*PGI and *Hs*PGI. (A) Structure of *Af*PGI in complex with Glc6P (orange sticks). (B) The apo structure of *Af*PGI. Glc6P (orange sticks) was placed into the active site by superimposing the structure of apo *Af*PGI onto that of *Af*PGI-Glc6P complex. The structure of *Hs*PGI in complex with erythose-4-phosphate (PDB code 1IRI). Download FIG S5, PDF file, 0.5 MB. Copyright © 2022 Zhou et al. 2022 Zhou et al. https://creativecommons.org/licenses/by/4.0/ This content is distributed under the terms of the Creative Commons Attribution 4.0 International license.

Since Ala221 is proximal to the phosphate-binding loop, to understand whether the enzyme activity can be affected through Ala221, an Ala221Gln mutant was introduced into *Af*PGI. A kinetic assay shows that the enzyme activity of Ala221Gln is the same as that of wild-type *Af*PGI (see Fig. S6), suggesting that it is difficult to directly inhibit the enzyme activity via Ala221. However, we unexpectedly observed that a citrate molecule (buffer component) binds to the gateway of the active site and is also recognized by the phosphate-binding loop in the apo *Af*PGI structure (Fig. 7C). The phosphate-binding loop adopts an “open” conformation compared to the structure of *Af*PGI-Glc6P complex and therefore places the citrate molecule proximal (5 Å) to Ala221, suggesting that the gateway between the active site and Ala221 is ligandable (Fig. 7D and E). Compounds bound to the gateway are potential starting points to explore specific fungal inhibitors. Taken together, these data suggest that the crystal structure of *Af*PGI reveals avenues for discovery of inhibitors.

10.1128/mbio.01426-22.6 FIG S6 Kinetics of *Af*PGI and *Af*PGI A221Q. The assay was carried out using a coupled G6PDH assay with Fru6P as the substrate. Error bars represents the SD of three determinations. Download FIG S6, PDF file, 0.1 MB. Copyright © 2022 Zhou et al. 2022 Zhou et al. https://creativecommons.org/licenses/by/4.0/ This content is distributed under the terms of the Creative Commons Attribution 4.0 International license.

## DISCUSSION

A. fumigatus is an opportunistic pathogen that causes hundreds of thousands of deaths each year (45, 46). To date, only a limited arsenal of antifungal agents has been approved for treatment of aspergillosis (47). Emerging resistance suggests an urgent need for novel classes of antifungal agents. Identification of new antifungal targets facilitates discovery of antifungal compounds. Acting as an extracellular skeleton armor for fungi, the cell wall is a dynamic organelle involved in not only protecting fungal cells from external stresses but also acting as virulence factors or pathogen-associated molecular patterns during host infection (48, 49). Since the cell wall is critical for fungal survival and virulence but is absent in humans, the cell wall and the enzymes involved in its biosynthesis are considered potential targets for antifungal drug development (10–13, 50). Currently, only one class of drug (echinocandins) targeting cell wall biosynthesis has been approved for clinical treatment of aspergillosis, suggesting that druggability of the fungal cell wall remains largely unexplored.

To identify new antifungal targets, we evaluated PGI in A. fumigatus as a potential candidate. Although biological functions of *pgi* have been extensively studied in various organisms, there is no study addressing the importance of *pgi* in A. fumigatus. Here, we have shown the first comprehensive study of *pgi* in A. fumigatus, which demonstrates that *pgi* is important for the survival of A. fumigatus, in agreement with previous studies in A. flavus and S. cerevisiae (15, 18). Through phenotypic analysis and direct determination of metabolic intermediates in A. fumigatus cells, we have demonstrated that PGI maintains the homeostasis of glycolysis, PPP and HBP. To our knowledge, our work shows the first evidence demonstrating that PGI plays a key role in maintaining the homeostasis of intracellular nucleotide sugars and phosphate sugars. Moreover, restoration of the viability by glucose/fructose also demonstrates that the enzymatic activity of PGI is important for A. fumigatus, which further suggests that PGI inhibitors could affect the growth of A. fumigatus. The growth defects of the Δ*pgi* strain could be partially rescued by galactose, GlcNAc, and glycerol. This phenomenon has also been observed in the Δ*pgi* strain of A. flavus (15), indicating the carbon metabolism connection between those sugars. The salvage or complementation pathway for this phenomenon requires further investigation.

Since we have demonstrated that PGI affects biosynthetic pathways of sugar nucleotides, the cell wall integrity is also likely to be affected by deleting *pgi*. Indeed, the cell wall integrity has been impaired in the Δ*pgi* mutant. Furthermore, previous studies in fungal cell wall have demonstrated that reduction of glucan is compensated by increased chitin (51–53). Our work shows that deletion of *pgi* also triggers this compensatory effect since glucan content is significantly reduced in the Δ*pgi* mutant (Fig. 4B). Notably, despite PGIs in A. fumigatus and A. flavus displaying similar functions in germination, conidiation and importance in virulence, remarkable differences were observed regarding to cell wall integrity and biosynthesis (15). The observed sensitivity to CAS and the altered glucan content in the A. fumigatus Δ*pgi* mutant was not detected in the A. flavus PGI-deficient mutant, suggesting that PGI plays different roles in cell wall biogenesis of close species. Although the cell wall of A. fumigatus is impaired by deleting *pgi*, the mutant in A. fumigatus was not sensitive to osmotic stabilizers, and the sporulation deficiency of the mutant could not be rescued (Fig. 4A), which is different from previous studies showing that PGI deficiency led to sensitivity to osmotic stabilizers in C. neoformans and F. oxysporum, and the sporulation defects of the A. nidulans *pgi* mutant were rescued by osmotic stabilizers (14, 19, 54). As such, the loss of *pgi* may have different effects in different fungal species. Taken together, our work suggests new insights regarding the functions of PGI for fungal species.

Although we have demonstrated that *pgi* is important for A. fumigatus by *in vitro* experiments, *in vivo* validation was executed showing reduced virulence of the Δ*pgi* mutant in nematode and larva infection models, which serves as the first evidence for the importance of *pgi* for A. fumigatus *in vivo*.

Because PGI is an important enzyme for the viability and pathogenicity of A. fumigatus, inhibitors against *Af*PGI are potential antifungal agents. As mentioned previously, the Δ*pgi* mutant is hypersensitive to CAS, an echinocandin-class drug for aspergillosis infections against which resistance is emerging widely (55). Therefore, inhibitors targeting *Af*PGI could be utilized together with CAS as a combinational therapy strategy for clinical treatment, especially for infections caused by echinocandin-resistant strains. Moreover, since humans and fungi are eukaryotes using similar cellular machineries, selectivity over human orthologues of targets is required for most antifungal agents. To date, PGI inhibitors are pseudo substrates harboring phosphate groups and thus exhibit low membrane permeability. To identify new types of inhibitors, we solved the first crystal structure of *Af*PGI in complex with Glc6P. The *Af*PGI structure suggests potential avenues for developing inhibitors lacking phosphate groups. A key structural difference with the *Hs*PGI active site groove suggests potential avenues for evolving selective inhibitors.

In summary, we have genetically and structurally validated PGI as a novel antifungal target against A. fumigatus. Our work provides a platform for development of small molecule compounds inhibiting the growth of A. fumigatus.

## MATERIALS AND METHODS

### Strains and culture conditions.

The A. fumigatus Ku80Δ*pyrG* strain was used as the parental strain for transformation, and the Ku80Δ strain was used as the WT for phenotypic analysis. Ku80Δ and revertant strains were cultured on complete medium (CM) and minimal medium (MM) (56), and 5 mM uridine and uracil were added when culturing Ku80Δ*pyrG*, whereas 1% (wt/vol) fructose was supplemented for the Δ*pgi* mutant. Conidia were collected with 0.2% (vol/vol) Tween 20 in double-distilled H_2_O. Mycelia were incubated in a liquid medium at 37°C for 48 h with shaking at 200 rpm and then washed thoroughly by distilled water, flash-frozen in liquid nitrogen, and stored at –80°C. Whenever required, the frozen mycelia were ground by mortar and pestle for DNA, RNA, and protein extraction.

### Construction of the Δ*pgi* mutant and revertant strains.

The Δ*pgi* mutant and revertant strains were constructed by homologous recombination. To generate the Δ*pgi* mutant, the upstream flanking region before the *pgi* start codon and the downstream flanking region after the stop codon were amplified with two pairs of primers—P11/P12 and P13/P14—and cloned into the modified PMD20T plasmid, which was digested by the FseI-PacI and AscI-NotI, respectively. The fragment *neo*-*pyrG-neo* (8.6 kb) was obtained from pCDA14 plasmid with HpaI digestion and cloned onto SmaI-digested PMD20T-up-down to generate the PMD20T-*pgi*. After linearizing via AscI and PacI sites, the *pgi* deletion fragment was transformed into KU80Δ*pyrG* protoplasts. Transformants were screened on MM supplemented with 1 M sorbitol and 1% (wt/vol) fructose, and then PCR was confirmed using the primer pairs P1/P2, P3/P4, P5/P6, and P7/P8.

To construct a revertant strain, the fragment of the upstream flanking region and integral *pgi* gene (2,855 bp from positions −1000 to +1855) was amplified with the primers P15/P16, the fragment of *AfpyrG* (1,891 bp) was amplified by the primers P17/P18, and the downstream flanking region (800 bp) was amplified by the primers P19/P20. These three fragments were purified and assembled in a pCE-Zero vector. The Δ*pgi* mutant was inoculated on CMU medium containing 1% (wt/vol) fructose, 0.1% (wt/vol) glucose, and 1 mg mL^−1^ 5-fluoroorotic acid to generate the Δ*pgi *Δ*pyrG* mutant. The assembled fragment amplified by primers P15/P20 was transformed into Δ*pgi *Δ*pyrG* protoplasts. All constructed strains were first confirmed by PCR and then by Southern blotting, in which NcoI and BglII were used for genomic DNA digestion; the *AnpyrG* and downstream regions were used as probes.

### Analysis of the Δ*pgi* mutant.

Serially diluted conidia starting from 10^5^ were spotted on solid MM supplemented with different concentrations of glucose, fructose, gluconate, and other carbon sources to test the growth of the WT, Δ*pgi*, and RT strains. Radial growth rates were measured by spotting 2 × 10^5^ conidia at the center of the plate and recording the colony diameter every 12 h. After 7 days, conidia were collected and counted by using a hemocytometer. Germination in liquid MMFG was conducted by placing coverslips at the bottom of the plate and inspecting them under the microscope at a specific time. The serial diluted conidia starting from 10^5^ were spotted on MMFG containing the following compounds: 50 μg mL^−1^ SDS, 50 μg mL^−1^ Congo red, 100 μg mL^−1^ Calcofluor White, 1.2 M sorbitol, 2 μg mL^−1^ itraconazole, 0.1 μg mL^−1^ voriconazole, 256 μg mL^−1^ fluconazole, 2 μg mL^−1^ caspofungin, and 1 μg mL^−1^ amphotericin B. All of these plates were cultured at 37°C for 48 h and photographed.

For quantitative analysis of cell wall monosaccharides, 10^8^ conidia were inoculated in 100 mL of liquid MMFG, followed by incubation at 37°C with shaking at 200 rpm for 48 h. Mycelia were collected, rinsed with deionized water, and frozen at –80°C. Cell wall extraction, polysaccharide hydrolysis, and quantification were conducted as described previously (57). The amounts of α-glucan and β-glucan were estimated by measuring released glucose using the phenol/sulfuric acid method (58). Chitin content was determined by measuring the glucosamine released after acid hydrolysis using a published method (59). Monosaccharides from the glycoprotein were separated on a CarboPac PA-10 column. Elution was performed at room temperature with a flow rate of 1 mL min^−1^ for 18 mM NaOH.

Virulence tests in C. elegans and G. mellonella models were conducted following a procedure described previously (28, 60).

### Analysis of intracellular metabolites.

Intracellular metabolites were extracted with chloroform/methanol buffer for phosphate sugars and nucleotide sugars according to the procedure described before (57). Next, 200 μL of metabolite extraction was used for the measurement of sugar phosphates and nucleotide sugars, respectively. Quantification of phosphate sugars followed a published method (57). Nucleotide sugars were analyzed on CarboPac PA-1 column with modifications as previously described (61), and using the following elution program: 100% solution A for 1 min; an elution gradient for 45 min with 90% solution A and 10% solution B; and an elution gradient of 10 min until 100% solution B is reached (solution A, 20 mM Tris-HCl [pH 9.2]; solution B, 2 M NaCl). The flow rate was 1 mL min^−1^.

### Expression and purification of *Af*PGI.

The A. fumigatus *pgi* gene was amplified from total cDNA by the primers P49/P50. This fragment was then cloned into plasmid pGEX-6P-1 by infusion assembly, yielding the plasmid pGEX-*Af*PGI, which was sequenced by GENEWIZ. To express proteins, the above plasmid was transformed into E. coli BL21(DE3)/pLysS and cultured in Luria-Bertani (LB) medium supplemented with 0.1 mg mL^−1^ ampicillin at 37°C. Then, 10 mL of overnight culture was inoculated into 1 L of LB medium with 0.1 mg mL^−1^ ampicillin and grown until it reached an optical density at 600 nm of 0.8. Protein expression was induced by 250 μM isopropyl-β-d-thiogalactopyranoside (IPTG), cultured at 17°C for 36 h, and harvested by centrifugation at 3,500 rpm and 4°C for 30 min. The cells were resuspended in a 25 mL of buffer (25 mM HEPES [pH 7.5], 150 mM NaCl, 1 mM EDTA, 10 mg mL^−1^ DNase, 0.5 mg mL^−1^ lysozyme, protease inhibitor) and disrupted using a sonicator on ice. After centrifugation at 12,000 rpm for 30 min at 4°C, the supernatant was incubated with preequilibrated glutathione beads on a rotating platform at 4°C for 2 h. The bound fusion protein was cleaved by precision protease to remove the GST tag at 4°C for 2 h. The eluted solution was concentrated to 2 mL and separated by using a Superdex 75 column (Amersham Bioscience) at a flow rate of 1 mL min^−1^. The collected protein was confirmed by 10% SDS-PAGE, and pure protein was concentrated to 10 mg mL^−1^ using a 10-kDa cutoff concentrator.

### Steady-state kinetics.

The activity of *Af*PGI for the reverse reaction was determined by coupling with glucose-6-phosphate dehydrogenase (G6PDH) in a reaction mixture containing 50 mM HEPES (pH 7.5), 2.5 mM MgCl_2_, 0.5 mM NADP^+^, 1 mM EDTA, 0.5 U of G6PDH, and Fru6P in a final volume of 0.2 mL and incubated at 30°C for 5 min. The concentrations of Fru6P used were 0.1, 0.3, 0.5, 0.75, 1, 1.5, 2.5, and 5 mM. NADPH production was monitored by determining the emission at 460 nm and excitation at 340 nm. One unit of enzyme is defined as 1 mM NADP^+^ reduced per minute under the experimental conditions.

The activity of *Af*PGI for the forward reaction was directly determined by HPAEC-PAD. In total, 0.2-mL reaction mixtures in 50 mM HEPES (pH 7.5), 2.5 mM MgCl_2_, 1 mM EDTA, and Glc6P were prepared, followed by incubation at 30°C for 20 min and then 100°C for 10 min to inactivate the enzymes. The concentrations of Glc6P used were 0.1, 0.3, 0.6, 1, 2, 5, 7.5, and 15 mM. Two times the volume of ethanol then was used to precipitate proteins at –20°C for 1 h. After centrifugation at 12,000 rpm for 10 min, the samples were loaded onto a CarboPac PA-1 column for measurement using the same program used for sugar phosphates. The results are presented as the means ± the standard deviations (SD) from three determinations.

### Crystallization and structure determination.

Finally, 10 mg/mL of *Af*PGI was incubated with or without 5 mM Glc6P for 2 h on ice before setting up crystal trays using sitting-drop vapor diffusion. Crystals appeared after 2 days in 0.1 M Na-HEPES (pH 7.5)–1.4 M tri-sodium citrate. Using a mixture of 85% mother liquor and 15% glycerol as cryoprotectant, the crystals were flash-frozen in liquid nitrogen. For crystals obtained with Glc6P, X-ray diffraction data were collected at the European Synchrotron Radiation Facility (ESRF; France) at 100K and autoprocessed using XDS software (62). The structure was solved by molecular replacement using pig PGI (PDB 1GZD) as the search model. Manual model building/refinement was applied using Coot (63), REFMAC5 (64), and other CCP4 software packages (65). For crystals obtained without Glc6P, X-ray diffraction data were collected at the Diamond Light Source (UK). Data were processed by using autoPROC (66). The phase problem was solved by molecular replacement using the *Af*PGI-Glc6P complex structure as the search model. Refinement was carried out using PHENIX (67). The final figures were made using PyMOL (68).

### Data availability.

The atomic coordinates and structure factors of *Af*PGI were deposited in the Protein Data Bank under accession codes 7OYL and 7U34.
