# Supplementary material for: Phosphoglucose Isomerase Is Important for Aspergillus fumigatus Cell Wall Biogenesis
Source: mBio. 2022 Aug 1;13(4):e01426-22. doi: 10.1128/mbio.01426-22 (PMC9426556; doi:10.1128/mbio.01426-22)
Supplement: FIG S1 [file mbio.01426-22-s0001.pdf]

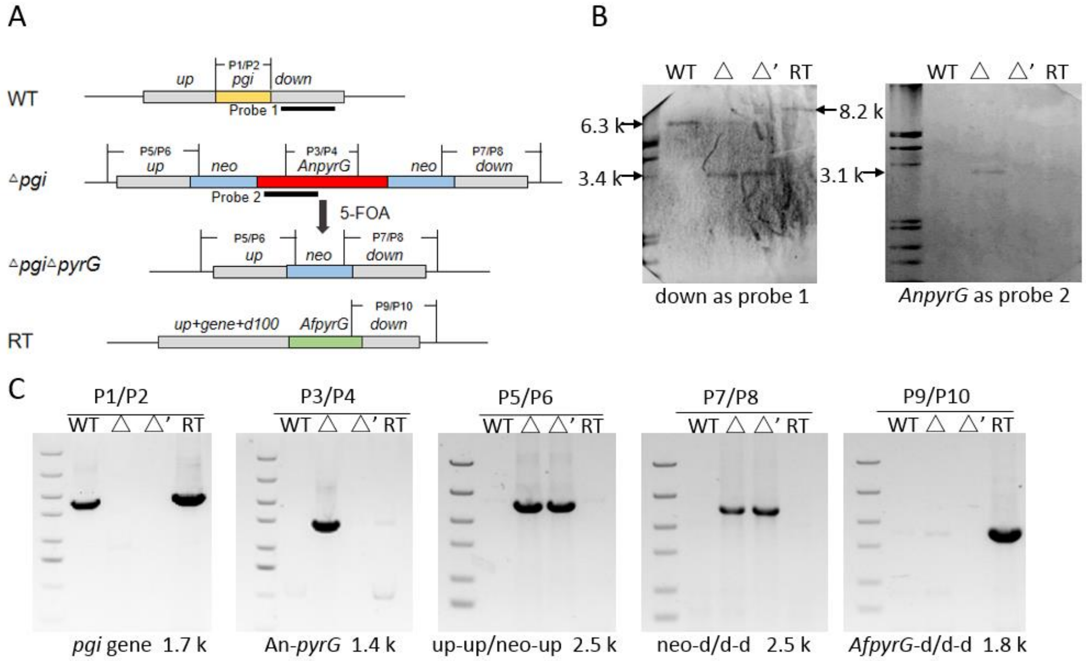

Fig. S1 Generation of the Δ*pgi* mutant.

A. Diagram illustrating strategies of constructing the Δ*pgi* mutant and revertant strains.

B. Southern blot analysis using total genomic DNA digested with *Bgl*II and *Nco*I and probed with downstream fragment of *pgi* and *AnpyrG*. The Δ*pgi*Δ*pgrG* strain was simply labeled as Δ'.

C. Confirmation of the mutant and revertant strains by PCR using primers P1/P2, P3/P4, P5/P6, P7/P8 and P9/P10 as shown in the figure. The Δ*pgi*Δ*pgrG* strain was labeled as Δ'.
