# Supplementary material for: Phosphoglucose Isomerase Is Important for Aspergillus fumigatus Cell Wall Biogenesis
Source: mBio. 2022 Aug 1;13(4):e01426-22. doi: 10.1128/mbio.01426-22 (PMC9426556; doi:10.1128/mbio.01426-22)
Supplement: FIG S2 [file mbio.01426-22-s0002.pdf]

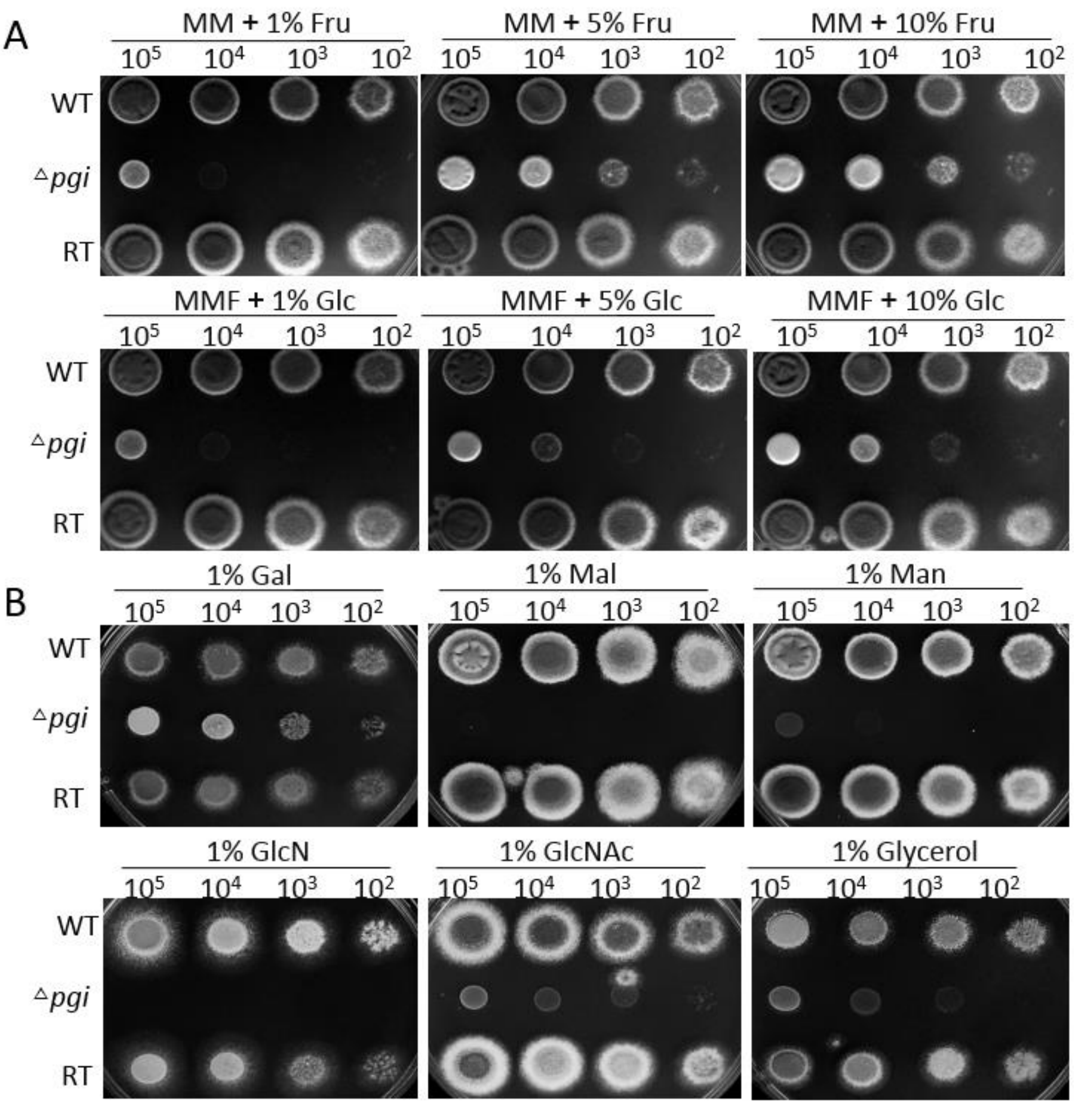

Fig. S2 Growth of the Δ*pgi* mutant in different carbon sources.

A. 10^2-10^5 conidia of the WT, Δ*pgi* and RT strains were inoculated onto MM medium with combinations of 1% Glc with 1% to 10% Fru, or 1% Fru with 1% to 10% Glc. Plate photos were taken after cultivation at 37 ℃ for 48 h.

B. Sole carbon sources such as 1% galactose (Gal), 1% maltose (Mal), 1% mannose (Man), 1% glucosamine (GlcN), 1% N-acetylglucosamine (GlcNAc) or 1% glycerol were used for growth tests. Plate photos were taken after cultivation at 37 ℃ for 48 h.
