# Supplementary material for: Phosphoglucose Isomerase Is Important for Aspergillus fumigatus Cell Wall Biogenesis
Source: mBio. 2022 Aug 1;13(4):e01426-22. doi: 10.1128/mbio.01426-22 (PMC9426556; doi:10.1128/mbio.01426-22)
Supplement: TABLE S1 [file mbio.01426-22-s0007.pdf]

Table S1 Primers used in this study

Primers	Primer sequences (5'-3')	Length (bp)
**Mutant construction**		
Pgi-up-F (P11)	CGTTAATTAAGCCCGAAACACAACTTAGGA	1301
Pgi-up-R (P12)	TATGGCCGGCCTGTGACCGCTGTGTAGGTTG	
Pgi-down-F (P13)	AAATATGCGGCCGCATCTTGTTGCCAACATTGACT	1086
Pgi-down-R (P14)	TT GGCGCGCCTGTTTTCGCTCTTCTTGGCA	
**Revertant construction**		
CR-*Af*-up-F (P15)	aattcggatcttccagagatAGACTTCTACCATTACTCTTTTACCTTTTAA	2855
CR-*Af*-up-R (P16)	ttgaggcTTGTGTGCTATGGAAGAGAAGAAGC	
CR-*AfpyrG*-F (P17)	ccatagcacacaaGCCTCAAACAATGCTCTTCACC	1891
CR-*AfpyrG*-R(P18)	catttcacGTCTGAGAGGAGGCACTGATGC	
CR-*Af*-down-F (P19)	ctcctctcagacGTGAAATGAAAGTCATCAGTTAAATATTG	800
CR-*Af*-down-R (P20)	ttcaactgccgttcgacgatCCGTAGCGGAGTGCAAGCG	
**Verification of the strains**		
Pgi-F (P1)	ATGCCTGGTTTCTCGCAGGC	1755
Pgi-R (P2)	TTACGCCAGGTTGGCCTTCT	
AnpyrG-2-F (P3)	GGATAGTAGCTGCATGGATCAACCCTT	1400
AnpyrG-2-R (P4)	ATGTCCTCCAAGTCGCAATTGACCTAC	
Pgi-upup-F (P5)	ACATTGAAGAGACGAATAGGCA	2489
neo-up-500R (P6)	TGGAAGAATTTGTCCACTACG	
neo-down-500F (P7)	GCGGGGATCTCATGCTGGAG	2501
Pgi-dd-R (P8)	TTAGGGGACTGTGGGCGAGA	
AfpyrG-D-F (P9)	ATATGGCCAGAGTATGCGGC	1800
Pgi-D-D-R (P10)	GCCCTCAACCCAGCTAACAT	
**Protein expression**		
Pgi-ORF-F (P49)	GGATCC ATGCCTGGTTTCTCGCAGGCTACG	1659
Pgi-ORF-R (P50)	GCGGCCGC TTACGCCAGGTTGGCCTTCTTCTT	
