# Supplementary material for: Phosphoglucose Isomerase Is Important for Aspergillus fumigatus Cell Wall Biogenesis
Source: mBio. 2022 Aug 1;13(4):e01426-22. doi: 10.1128/mbio.01426-22 (PMC9426556; doi:10.1128/mbio.01426-22)
Supplement: TABLE S2 [file mbio.01426-22-s0008.pdf]

Table S2. Germination rate of the three strains in liquid MMFG.

Time (h)	Germination rate		
	WT	Δ*pgi*	RT
8	97 ±1	40 ±9	97 ±1
9	-	47 ±6	-
10	-	54 ±6	-
11	-	65 ±7	-
12	-	72 ±6	-

10^5 conidia were incubated into liquid MMFG at 37 ℃ cultivation for the indicated time. The germination rate from one hundred conidia of each strain was calculated, and the experiment was repeated three times. Values represent the mean ±*SD*, - denotes full germination.
