# Supplementary material for: Phosphoglucose Isomerase Is Important for Aspergillus fumigatus Cell Wall Biogenesis
Source: mBio. 2022 Aug 1;13(4):e01426-22. doi: 10.1128/mbio.01426-22 (PMC9426556; doi:10.1128/mbio.01426-22)
Supplement: TABLE S3 [file mbio.01426-22-s0009.pdf]

Table S3 Monosaccharides and intracellular sugars in the three strains.

Table S3A Monosaccharides released from cell wall glycoproteins in the three strains.

Strains	glycoprotein			
	Protein (μg)	GlcNAc (μg)	Gal (μg)	Man (μg)
WT	20.20 ± 3.14	6.47 ± 1.28	1.84 ± 0.56	1.82 ± 0.59
Δ*pgi*	285.35 ± 18.08	1.78 ± 0.34	0.20 ± 0.09	2.84 ± 0.87
RT	19.52 ± 1.88	6.32 ± 0.87	1.65 ± 0.51	1.58 ± 0.30

10^8 conidia were inoculated in 100 ml liquid MMFG medium and incubated at 37 ℃ for 48 h, then the mycelia were harvested for cell wall analysis. Monosaccharides from cell wall glycoproteins were released by acid hydrolysis, then were quantified by HPAEC-PAD. Values represent the mean ± *SD* from three replicates, and multiple *t*-tests were applied to calculate *P* values.

Table S3B Intracellular nucleotide sugars in the three strains.

Strains	Nucleotide sugar		
	UDP-GlcNAc (nmol)	UDP-Glc (nmol)	GDP-Man (nmol)
WT	124.47 ± 19.15	14.48 ± 0.81	4.20 ± 1.22
Δ*pgi*	354.85 ± 22.39	3.40 ± 1.79	13.65 ± 5.32
RT	140.95 ± 31.03	14.48 ± 3.21	5.07 ± 1.44

10^8 conidia were inoculated in 100 ml liquid MMFG medium and incubated at 37 ℃ for 48 h, then the mycelia were harvested for nucleotide sugar extraction. Three 50 mg dried mycelia were used for nucleotide sugar analysis. Three biological replicates were performed and multiple *t*-tests were applied to calculate *P* values. Values represent the mean ± *SD*.

Table S3C Intracellular phosphate sugars in the three strains.

Strains	Phosphate sugar				
	Glc6P (nmol)	Man6P (nmol)	Fru6P (nmol)	FBP (nmol)	6PG (nmol)

WT	6.88 ±0.19	2.35 ±0.06	11.78 ±0.20	16.42 ±1.55	1.23 ±0.26
Δ*pgi*	0.66 ±0.01	9.29 ±0.59	15.32 ±1.34	27.16 ±2.02	0.37 ±0.14
RT	6.89 ±0.22	1.91 ±0.95	11.07 ±1.18	10.21 ±0.42	0.93 ±0.10

10^8 conidia were inoculated in 100 ml liquid MMFG medium and incubated at 37 ℃ for 48 h, then the mycelia were harvested for phosphate sugar extraction. Three 50 mg dried mycelia were used for phosphate sugar analysis. Three biological replicates were performed and multiple *t*-tests were applied to calculate *P* values. Values represent the mean ±*SD*.
