# Supplementary material for: Phosphoglucose Isomerase Is Important for Aspergillus fumigatus Cell Wall Biogenesis
Source: mBio. 2022 Aug 1;13(4):e01426-22. doi: 10.1128/mbio.01426-22 (PMC9426556; doi:10.1128/mbio.01426-22)
Supplement: TABLE S4 [file mbio.01426-22-s0010.pdf]

Table S4 Survival rate of the nematodes and *G. mellonella* larvae infected by the indicated strains.

Table S4A Survival rate and hyphal filamentation rate of *glp-4*(bn2); *sek-1*(km4) worms infected by the indicated strains.

Strain	Survival rate			Hyphal filamentation at 24 h (%)	Worm numbers
	24 h	48 h	72 h		
OP50	99 ±1	98 ±1	98 ±2	0	1009
WT	50 ±6	21 ±5	16 ±2	64 ±4	1174
Δ*pgi*	93 ±4	75 ±3	63 ±4	16 ±8	1204
RT	71 ±3	33 ±6	24 ±5	45 ±3	1211

Three biological repeats (each with triplicates) were performed and total counted worm numbers are indicated.

Table S4B Survival rate of *G. mellonella* larvae infected by the indicated strains.

Time （h）	WT	Δ *pgi*	RT	0.02% Tween 20
24	74 ± 2	80 ± 3	71 ± 8	88 ± 4
48	67 ± 3	78 ± 7	57 ± 9	84 ± 5
72	41 ± 10	70 ± 9	39 ± 7	84 ± 5

Tween 20 inoculated larvae were used as the control to ensure that environmental conditions and physical injuries by inoculation did not affect the survival rates.
