# Supplementary material for: Phosphoglucose Isomerase Is Important for Aspergillus fumigatus Cell Wall Biogenesis
Source: mBio. 2022 Aug 1;13(4):e01426-22. doi: 10.1128/mbio.01426-22 (PMC9426556; doi:10.1128/mbio.01426-22)
Supplement: FIG S3 [file mbio.01426-22-s0003.pdf]

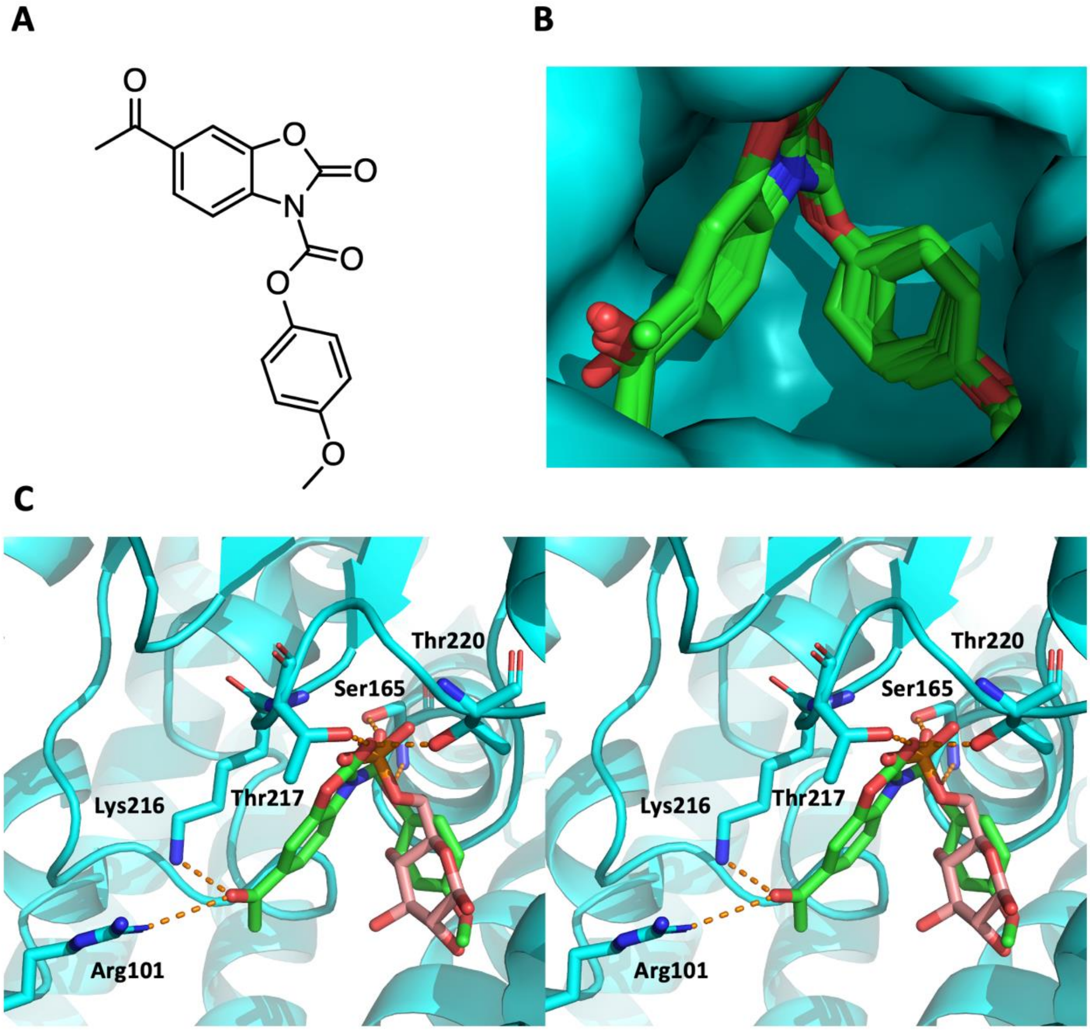

Fig. S3 Prediction of the binding mode of a neutral bisphosphate mimic in the active site of *Af*PGI.

A. The structure of the neutral bisphosphate mimic.

B. Predicted binding mode of the neutral bisphosphate mimic (green sticks) in the *Af*PGI active site (cyan). Prediction was carried out by AutoDock using the *Af*PGI-Glc-6P structure as the macromolecule for docking.

C. Representative binding mode of the neutral bisphosphate mimic. Orange dots indicate hydrogen bonds.
