# Supplementary material for: Phosphoglucose Isomerase Is Important for Aspergillus fumigatus Cell Wall Biogenesis
Source: mBio. 2022 Aug 1;13(4):e01426-22. doi: 10.1128/mbio.01426-22 (PMC9426556; doi:10.1128/mbio.01426-22)
Supplement: FIG S4 [file mbio.01426-22-s0004.pdf]

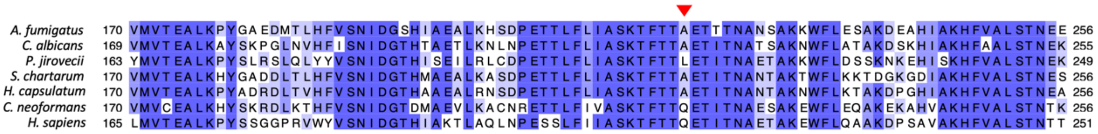

Fig. S4 Partial sequence alignment of PGIs. Red triangle indicates Ala221 in *Af*PGI. Sequences are from GenBank with codes as EDP54506.1 (*A. fumigatus*), XP_713513.1 (*Candida albicans*), XP_018229466.1 (*Pneumocystis jirovecii*), KEY64212.1 (*Stachybotrys chartarum*), QSS62647.1 (*Histoplasma capsulatum*), XP_569228.1 (*Cryptococcus neoformans*), NP_000166.2 (*Homo sapiens*).
