# Supplementary material for: Phosphoglucose Isomerase Is Important for Aspergillus fumigatus Cell Wall Biogenesis
Source: mBio. 2022 Aug 1;13(4):e01426-22. doi: 10.1128/mbio.01426-22 (PMC9426556; doi:10.1128/mbio.01426-22)
Supplement: FIG S5 [file mbio.01426-22-s0005.pdf]

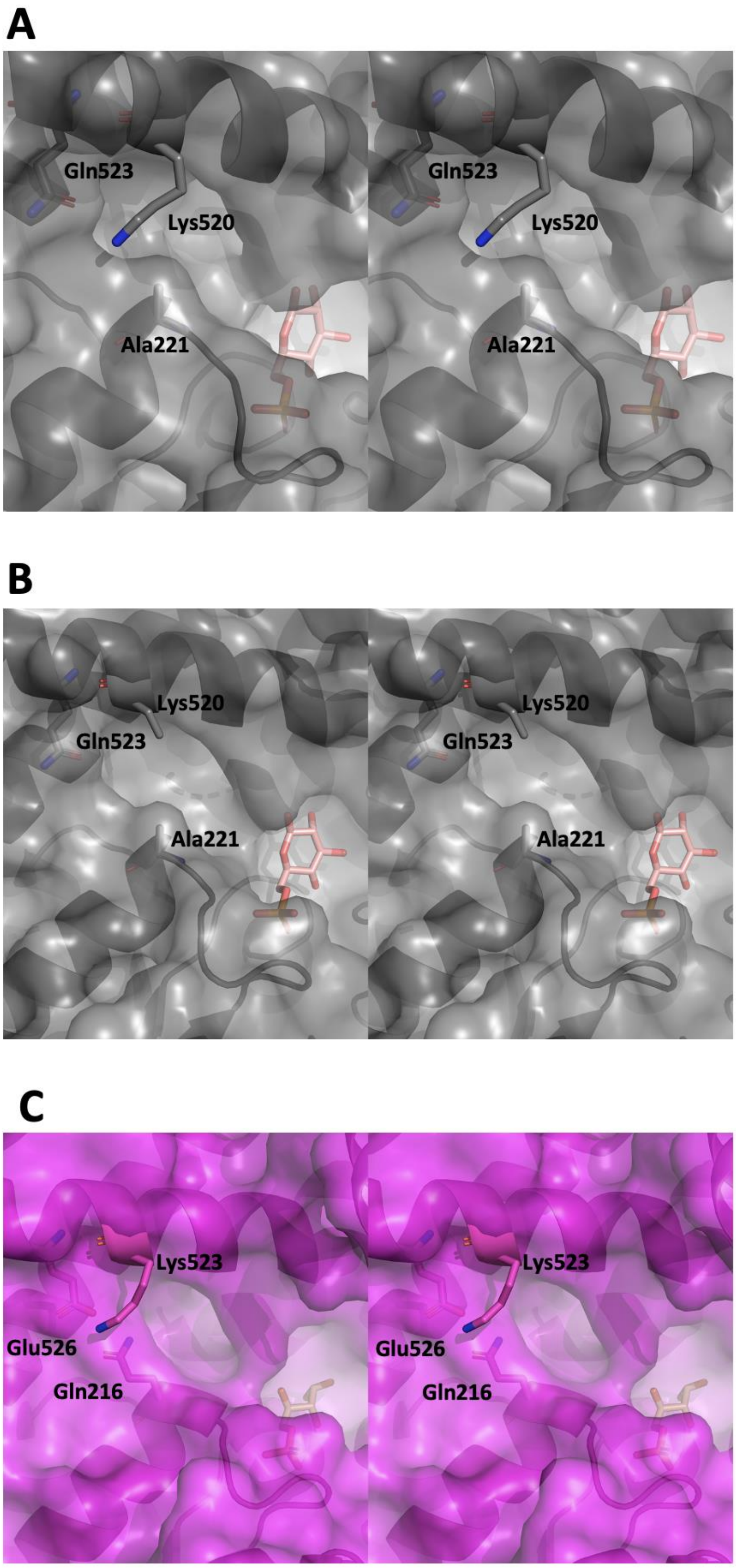
A
Gln523
Lys520
Ala221
B
Lys520
Gln523
Ala221
C
Lys523
Glu526
Gln216

Fig. S5 Structures of *Af*PGI and *Hs*PGI.

A. The structure of *Af*PGI in complex with Glc6P (orange sticks).

B. The apo structure of *Af*PGI. Glc6P (orange sticks) was placed into the active site by superimposing the structure of apo *Af*PGI onto that of *Af*PGI-Glc6P complex.

C. The structure of *Hs*PGI in complex with erythose-4-phosphate (PDB code 1IRI).
