# Supplementary material for: Phosphoglucose Isomerase Is Important for Aspergillus fumigatus Cell Wall Biogenesis
Source: mBio. 2022 Aug 1;13(4):e01426-22. doi: 10.1128/mbio.01426-22 (PMC9426556; doi:10.1128/mbio.01426-22)
Supplement: FIG S6 [file mbio.01426-22-s0006.pdf]

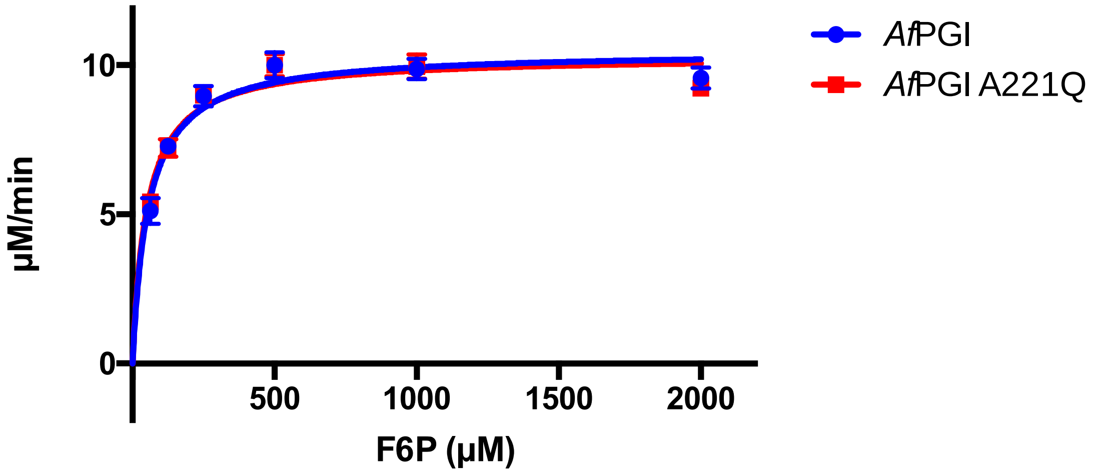

Fig. S6 Kinetics of *Af*PGI and *Af*PGI A221Q. The assay was carried out using a coupled G6PDH assay with Fru6P as substrate. Error bars represents standard deviation of three determinations.
